# Statistical-based database fingerprint: chemical space dependent representation of compound databases

**DOI:** 10.1186/s13321-018-0311-x

**Published:** 2018-11-22

**Authors:** Norberto Sánchez-Cruz, José L. Medina-Franco

**Affiliations:** Department of Pharmacy, School of Chemistry, Universidad Nacional Autónoma de México, Avenida Universidad 3000, 04510 Mexico City, Mexico

**Keywords:** Chemical space, Epi-informatics, Molecular fingerprints, Representation, Similarity searching

## Abstract

**Background:**

Simplified representation of compound databases has several applications in cheminformatics. Herein, we introduce an alternative and general method to build single fingerprint representations of compound databases. The approach is inspired on the previously published modal fingerprints that are aimed to capture the most significant bits of a fingerprint representation for a compound data set. The novelty of the herein proposed statistical-based database fingerprint (SB-DFP) is that it is generated based on binomial proportions comparisons taking as reference the distribution of “1” bits on a large representative set of the chemical space.

**Results:**

To illustrate the Method, SB-DFPs were constructed for 28 epigenetic target data sets retrieved from a recently published epigenomics database of interest in probe and drug discovery. For each target data set, the SB-DFPs were built based on two representative fingerprints of different design using as reference a data set with more than 15 million compounds from ZINC. The application of SB-DFP was illustrated and compared to other methods through association relationships of the 28 epigenetic data sets and similarity searching. It was found that SB-DFPs captured overall, the common features between data sets and the distinct features of each set. In similarity searching SB-DFP equaled or outperformed other approaches for at least 20 out of the 28 sets.

**Conclusions:**

SB-DFP is a general approach based on binomial proportion comparisons to represent a compound data set with a single fingerprint. SB-DFP can be developed, at least in principle, based on any fingerprint and reference data set. SB-DFP is a good alternative for exploration of relationships between targets through its associated compound data sets and performing similarity searching.

**Electronic supplementary material:**

The online version of this article (10.1186/s13321-018-0311-x) contains supplementary material, which is available to authorized users.

## Background

Molecular fingerprints are bit strings representations of chemical structures in which each position indicates the presence (1) or absence (0) of chemical features as defined in the design of the fingerprint. There are several types of molecular fingerprints described elsewhere [1, 2]. Such representations are broadly employed for the assessment of chemical space coverage, molecular diversity and similarity searching [1–3]. With the constant increasing size of chemical databases, such studies have become more computationally demanding, leading to the need of generating simplified representations of compound databases to optimize storage and calculation speed. To this end, many of the approaches that have been proposed generate a single fingerprint trying to capture the common chemical features presents in all compounds in a database (or at least in most of them). The first strategy dates back to 1996, when Shemetulskis et al. [4] employed the Daylight Chemical Information Systems, Inc. molecular fingerprint to build the so-called modal fingerprint, which contains the common bits found in the molecular fingerprints in a given compound data set. In the modal fingerprint, the degree to which bits have to be in common in the data set in order to be set as “1” is determined by a user-defined threshold value, which ranges from 50 to 100%, being 50% the best performing threshold in different studies. Since 1996 the algorithm has been extended to different molecular fingerprints and a number of studies have shown its application in similarity searching [5, 6] and for the quantification of intra- and inter-database diversity [7]. In parallel, several modifications to this concept have been developed, mostly aiming to enhance its performance on similarity searches at the expense of increasing the complexity to implement the approach. Such approaches include bit scaling [8–10], bit silencing [11] and the determination of the best feature combinations [12]. In different publications the term “modal fingerprint” has been used to refer to distinct approaches. To avoid confusions, herein we refer as “database fingerprint (DFP)” to the modal fingerprint constructed using 50% as the predefined threshold.

In this work, we present the statistical-based database fingerprint (SB-DFP) as a novel and general approach to generate a compound database fingerprint based on binomial proportion comparisons. In this paper, we illustrate the application of SB-DFP in comparing target-associated compound data sets and performing similarity searching. As a case study, and to further advance the emerging field of epi-informatics [13], the SB-DFPs were applied to a recently published epigenomics database with potential therapeutic significance.

## Methods

### Concept and construction of SB-DFP

As commented on the Background, in a “classic” DFP representation, to set a bit “1” requires that such bit is present in at least 50% of all the molecules in the input data set. The basic idea of such threshold is to extract common bits in at least half of the input data set. However, the underlying hypothesis assumes that the probability of presence of a feature (bit) in a molecular representation is 50% for each of them, so all bits are compared against such probability.

**Fig. 1:**
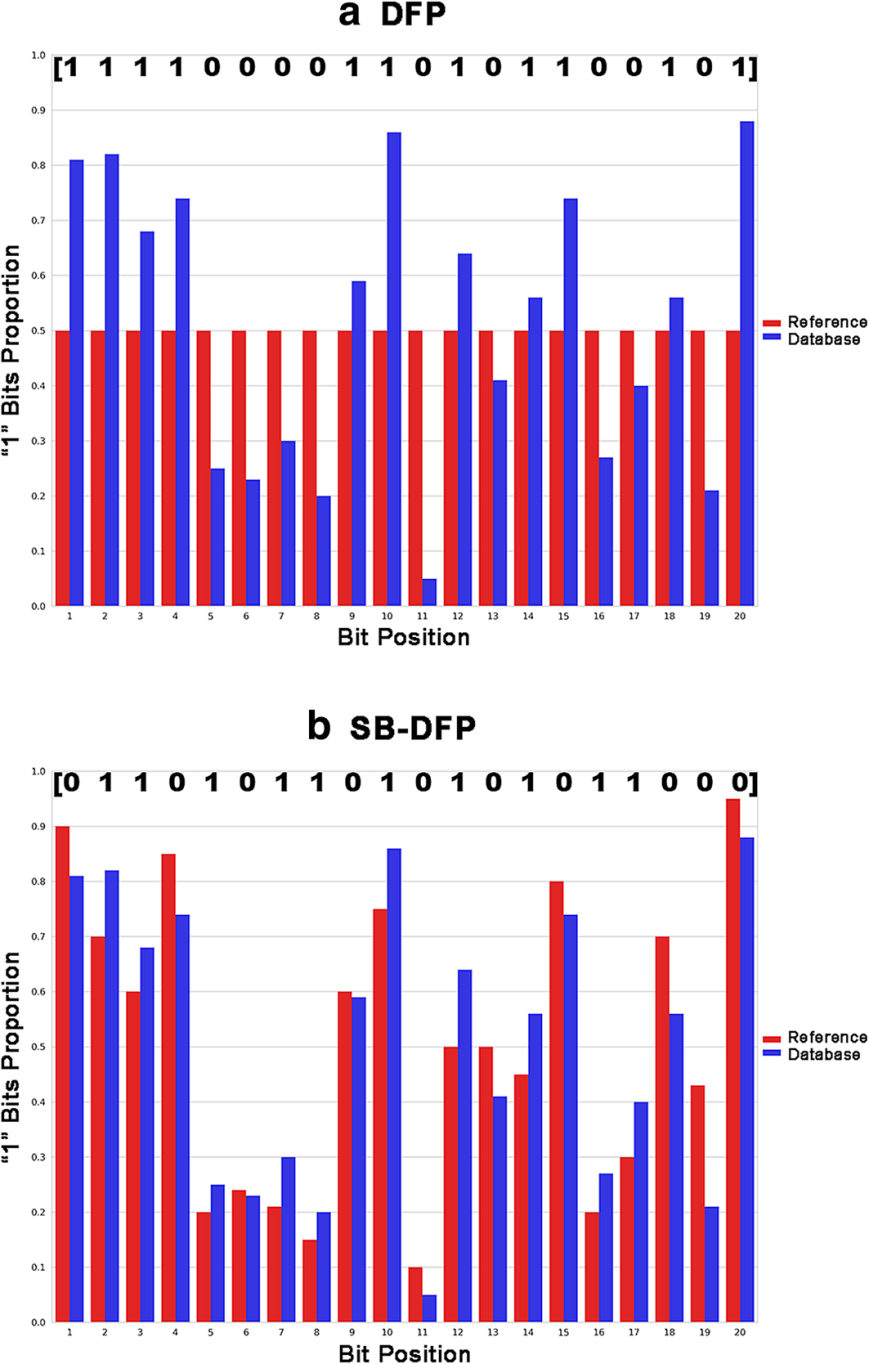
Schematic representation of single fingerprints for a compound database and an hypothetical 20-bit fingerprint. The upper part of charts shows the binary representation of the generated single fingerprint: **a** database fingerprint (DFP) and **b** statistical-based database fingerprint (SB-DFP)

SB-DFP is based on the basic hypothesis that the probability of presence of a feature (bit) in a molecular representation is not equal for each bit. Instead, it is determined by the availability of such feature in a reference set e.g. the “known chemical space” (or a reasonable approximation) and such availability has to be determined. Once the frequency occurrence of each bit in a molecular representation is determined for both, namely the reference set and the data set of study, the SB-DFP is constructed by comparing the frequency occurrence of each bit between both sets. Thus, a bit is set to “1” only if the frequency in the target data set is statistically higher than the reference. Figure 1 depicts a schematic comparison between a classic DFP (reminiscent of the modal fingerprint, vide supra) and the SB-DFP, respectively. In this scheme the database fingerprint is illustrated for a short hypothetical fingerprint representation with 20-bit positions.

It should be noted that the SB-DFP representation for a given data set requires three main features (Fig. 1b): (1) a reference set, (2) a molecular fingerprint representation and 3) a statistical method to do the binomial proportion comparisons. The chosen features for this work are described below, although SB-DFP can be developed with different fingerprints, reference sets, and statistical methods.

### Compound data sets

As a case study we generated SB-DFPs for a recently published epigenomics database [14]. The set of targets used as a test case in this work were selected based on their relevance in probe and epigenetic drug discovery that have attracted the attention to perform virtual screening [15, 16]. However, the SB-DFP is general and could be used for other targets. The epigenomics database used in this study contains compounds associations against 60 epigenetic targets. For our analysis, we selected the information for 28 targets for which there was at least 50 reported compounds with a potency of 10 µM or better. Table 1 summarizes the targets considered in this work that included bromodomain-containing proteins (BRD2, BRD3 and BRD4), histone acetyltransferases (CREBBP and EP300), DNA methyltransferase (DNMT1), histone lysine methyltransferase (EHMT2), histone deacetylases (HDAC1-HDAC11), lysine acetyltransferase (KAT2B), lysine demethylases (KDM1A and KDM4C), histone methyl-lysine binding proteins (L3MBTL1 and L3MBTL3), mitogen-activated protein kinase (MAP3K7), O-GlcNAcase (MGEA5), nuclear receptor coactivators with histone acetyltransferase activity (NCOA1 and NCOA3), and protein arginine methyltransferase (PRMT1). Table 1 also includes the number of compounds in each set (350 compounds on average with a maximum of 2740 for HDAC1). Note that SB-DFP could be applied to other data sets with larger number of compounds and their performance in, for instance, virtual screening, would need to be assessed in a case-by-case basis. It might be anticipated that the performance could be target-dependent as it happens in other virtual screening approaches.

**Table 1:** Selected datasets from the epigenomic database

Dataset	Number of compounds	Intra-set similarity median (Tc)		Average “1” bits		Number of “1” bits in DFP		Number of “1” bits in SB-DFP	
		MACCS^a^	ECFP4^b^	MACCS^a^	ECFP4^b^	MACCS^a^	ECFP4^b^	MACCS^a^	ECFP4^b^
BRD2	234	0.569	0.152	56.0	54.3	53	27	67	229
BRD3	246	0.573	0.153	56.6	54.6	53	26	73	231
BRD4	477	0.486	0.133	55.9	52.8	47	14	71	333
CREBBP	105	0.694	0.276	56.1	53.9	52	36	50	185
DNMT1	127	0.403	0.115	55.4	51.7	50	13	62	281
EHMT2	61	0.636	0.228	62.4	55.7	62	41	56	167
EP300	57	0.425	0.106	58.2	55.7	53	11	56	285
HDAC10	190	0.514	0.165	53.2	50.6	50	17	46	272
HDAC11	137	0.494	0.156	51.2	50.8	48	16	42	229
HDAC1	2740	0.453	0.149	53.2	51.4	51	15	63	499
HDAC2	767	0.447	0.149	50.3	48.4	46	13	53	336
HDAC3	669	0.474	0.147	52.6	50.3	49	13	54	356
HDAC4	452	0.427	0.135	50.4	46.4	42	10	49	248
HDAC5	112	0.455	0.153	47.3	44.1	39	13	26	176
HDAC6	1374	0.474	0.149	54.3	49.8	48	13	62	415
HDAC7	112	0.489	0.165	50.4	45.8	43	12	28	197
HDAC8	864	0.500	0.153	54.9	51.2	50	12	52	398
HDAC9	102	0.494	0.169	52.6	47.4	46	13	29	190
KAT2B	55	0.583	0.179	50.8	37.3	46	13	44	99
KDM1A	241	0.380	0.143	44.8	46.2	31	21	31	216
KDM4C	88	0.359	0.101	48.8	40.3	41	10	38	158
L3MBTL1	50	0.804	0.551	42.2	36.8	37	27	37	56
L3MBTL3	89	0.731	0.404	40.4	36.6	37	26	35	83
MAP3K7	96	0.539	0.137	57.1	60.5	59	35	45	190
MGEA5	67	0.683	0.316	54.2	39.6	48	19	42	126
NCOA1	51	0.350	0.105	45.5	43.3	34	11	18	132
NCOA3	157	0.368	0.109	47.7	44.6	39	10	26	166
PRMT1	61	0.395	0.076	53.0	53.5	41	9	40	239
Average	350	0.507	0.178	52	48	46	18	46	232

^a^MACCS keys 166-bit

^b^ECFP4 2048-bit

### Reference set

In this study, the All Clean subset from the ZINC12 database [17], with 16,403,844 unique compounds, was selected as starting point to build the reference set for SB-DFP calculations. We removed 21 compounds that could not be processed by the RDKit module for Python [18] and also 154 compounds present in the epigenomics database. The remaining molecules were randomly divided in two groups: one group with 1,000,000 compounds to be used as decoys in similarity searching (vide infra) and the second group with the remaining 15,403,690 molecules to be used as reference for SB-DFP calculations. We employed such database with more than 15 million compounds as a representative sample of the currently known chemical space of small molecules available in ZINC. We emphasize that SB-DFP could be implemented using other reference data sets.

### Fingerprints

We selected two fingerprints to illustrate the applicability of the concept of SB-DFP: Molecular ACCess System (MACCS) keys (166-bit) [19] as a “low resolution” dictionary fingerprint, and Extended Connectivity Fingerprint diameter 4 (ECFP4) as a “high resolution” representation [20] in its folded version of 2048 bits. MACCS keys and ECFP4 were generated with RDKit.

### Binomial proportion comparisons

To perform the binomial proportion comparisons we employed a Z-test, as implemented in the statsmodels [21] module for Python. As can be found elsewhere [22], the proportion comparison relies on the calculation of a test statistic (called $Z_{test}$) defined as:

$$Z_{test} = \frac{{p_{t} - p_{r} }}{{\sqrt {\varvec{P}\left( {1 - \varvec{P}} \right)\left( { \frac{1}{{n_{t} }} + \frac{1}{{n_{r} }} } \right)} }}$$

where $p_{t}$ and $p_{r}$ are the proportions in which a given bit appears as “1” in the target and reference data sets for a total of $n_{t}$ and $n_{r}$ observations, respectively. $\varvec{P}$ is the estimated true proportion of “1” bits considering both sample observations and it is calculated as:

$$\varvec{P} = \varvec{ }\frac{{n_{\varvec{t}} p_{t} \varvec{ } + \varvec{ }n_{\varvec{r}} p_{r} }}{{n_{t} + \varvec{ }n_{r} }}$$

With the $Z_{test}$ calculated and through the standard Normal distribution, the exact probability than the observed difference between proportion is due to random variation can be determined (the *p* value). So that the proportion difference is statistically significative if the *p* value is lower than the associated to the confidence level selected a priori. For example, for the bit 100 in MACCS fingerprint, the bit “1” occurrence in the reference set is 10,892,579 from 15,403,690 observations ($p_{r} = 0.707$). By selecting a confidence level of 99% (*p* value < 0.01) and doing the calculations one gets that for a target data set of 350 compounds, the bit occurrence must be equal or greater than 268 ($p_{t}$ = 0.766, *p* value = 0.008) to be set as an “1” bit in the SB-DFP representation even when for a bit occurrence of 248 the proportion seems to be larger ($p_{t}$ = 0.708, *p* value = 0.476). This example illustrates that a greater proportion of “1” in a given bit for the target data set in comparison to the reference data set does not necessarily implies that such bit will be set as “1” in the SB-DFP. In other words, the proportion difference must be “big enough”.

For this work we choose a confidence level of 99% (*p* value < 0.01) based on the average AUC values obtained from similarity searching for ECFP4 and MACCS keys at five different confidence levels (vide infra). For the sets of targets and the fingerprints explored, the best performing method is the one with a confidence level of 99% (Additional file 1: Table S1) and all further calculations and discussion are based on such method. Of note, other *p* values could be chosen for other targets and/or other fingerprints.

### SB-DFP to study inter-data set relationships

To evaluate the performance of SB-DFP to capture the differences between data sets we calculated both, the classic DFP and the SB-DFP for each of the 28 targets. Both database fingerprints were constructed based on ECFP4 and MACCS keys fingerprints. Using the Tanimoto coefficient [23] and for each molecular fingerprint, we constructed the similarity matrices between epigenetic targets with three methodologies to calculate the similarity between pairs of targets: the median similarity between all-compound comparisons (ACC) in the data sets, the similarity between DFPs, and the similarity between SB-DFPs. This led to a total of six representations herein referred as ACC/MACCS, ACC/ECFP4, DFP/MACCS, DFP/ECFP4, SB-DFP/MACCS and SB-DFP/ECFP4. The range of similarity values for each representation was taken as a measure of its resolution. All six similarity matrices were transformed to their corresponding distance matrices based on the relationship (distance = 1 − similarity). The distance matrices were used as basis for hierarchical clustering with complete linkage to analyze the ability of the representations to recover the known relationships between epigenetic targets based on its sequence identity. Such ability was assessed by calculating the Adjusted Rand Index (ARI) of each clustering [24] at a level of 10 clusters. The ARI measures the similarity between a given clustering and a ground truth: an ARI value of 1 indicates that the clustering recovers the original groups and an ARI value of 0 indicates random assignations. As ground truth, we used the hierarchical clustering with complete linkage obtained from the distance form of the sequence identity matrix (shown as Additional file 1: Table S10) as obtained from the alignment with Clustal Omega [25] with default parameters for the 28 targets studied. Sequences for all targets were taken from the Universal Protein Knowledgebase (UniProt) [26]. In addition, the number of “1” bits present in each representation was calculated as an approach of the amount of information contained in each one.

### SB-DFP as query for similarity searching

Previous studies have shown that using single fingerprint representation of compound databases as query yield better results in similarity searching than fingerprint representations of single compounds [5, 6]. However, when single fingerprint representations are compared with methods that use information for multiple compound in a database, such as k-nearest neighbors (k-NN) and binary kernel discrimination, the single fingerprint searches are outperformed [5]. In this work, we tested the performance of SB-DFP in similarity searching as compared to the classic DFP and 1-NN search strategies for both MACCS keys and ECFP4 fingerprints, methods such as binary kernel discrimination were not compared in this work given its reported lack of efficiency [5]. The Tanimoto coefficient was used as similarity measure, although other similarity metrics could be explored. For SB-DFP, five different confidence levels were tested for binomial proportion comparisons, here we report only the best performing one (99%), the rest are summarized in Additional file 1: Table S1.

Using an approach similar to the one reported by Heikamp et al. [27], from each of the 28 epigenetic targets, 100 random sets of 10 active compounds each were randomly selected and used as query. In each case, all remaining active compounds were added as active database of compounds (ADCs) to a database containing one million compounds randomly selected from the ZINC All Clean subset (vide supra), called the search set. For the searches involving DFP and SB-DFP, the 10 compounds used as query were employed to build the corresponding single fingerprint, which was compared against all compounds in the search set, leading directly to a single similarity value per compound. On the other hand, for 1-NN, each of the compounds in the search set was compared to the 10 compounds used as query, leading to 10 similarity values per compound, from which the highest value was taken. For each similarity search, the compound recovery rates (RR) were calculated in a target-specific selection over the number of available ADCs as a measure of early enrichment. Receiver operating characteristic (ROC) curves and ROC area under the curve (AUC) values were also computed.

## Results and discussion

### Bit proportions in the reference set

As detailed in the Methods section, 15,403,690 compounds from the ZINC All Clean subset were taken as a representative sample of the currently known chemical space of small molecules. For the complete data set, the frequency of each bit was calculated for ECFP4 and MACCS keys. The results are summarized in Additional file 1: Tables S2 and S3. Of note, only 43 out of 166 bits for MACCS keys and 12 out of 2048 bits for ECFP4 have frequencies over 0.5. This means that 43 and 12 bits of MACCS keys and ECFP4, respectively, are the most likely to appear in the DFP representation of any data set. Such bias is avoided in SB-DFP.

### Compound data sets

For the 28 data sets studied in this work a total of six representations were generated for each set: the fingerprints for each compound, the single DFP, and SB-DFP, all based on ECFP4 and MACCS keys, respectively. Of note, the data sets representations based on DFP and SB-DFP have the advantage over “all-compounds” representation in that the speed of calculation is *NxM* times faster than doing pairwise comparisons with all compounds in a set (with *N* and *M* being the number of compounds in two data sets).

The median of the intra-set similarity for all compounds in each data set was computed with MACCS keys and ECFP4 and the results are summarized in Table 1. Overall, all 28 sets have structural diverse compounds with, for instance, maximum median MACCS keys similarity of 0.694 (average of 0.507) and maximum median ECFP4 similarity of 0.551 (average of 0.178).

Table 1 also reports the average number of “1” bits for all compounds, as well as the number of “1” bits in the DFP and SB-DFP, respectively. For both MACCS keys and ECFP4 fingerprints, DFP representation has, on average, number of “1” bits (46 and 18, respectively) lower than all-compounds representation (52 and 48, respectively) but higher than the number of bits with occurrence frequencies over 0.5 in the reference set (vide supra). As expected, DFP contains less information than the complete data set. However, DFP captures more features in the data set than expected according to the occurrence frequencies in the reference data set.

DFP/MACCS and SB-DFP/MACCS capture similar amount of information with an average number of “1” bits of 46. However, as shown in Table 1, there is a dramatic increase in the number of “1” bits for SB-DFP/ECFP4 as compared to DFP/ECFP4 (232 vs. 18). These results indicate that for the 28 data sets considered in this work, SB-DFP/ECFP4 captures a higher amount of specific structural features of the compounds.

### Similarity matrices

The similarity matrices between epigenetic targets were calculated with three different approaches to calculate the similarity between pairs of targets: the median similarity of the all pairwise comparisons (e.g., all-compound comparisons) in the data sets (ACC), the similarity between their DFPs, and the similarity between their SB-DFPs, all based on MACCS keys and ECFP4 using the Tanimoto coefficient. As described in the Methods section, these representations are referred in this work as ACC/MACCS, ACC/ECFP4, DFP/MACCS, DFP/ECFP4, SB-DFP/MACCS, and SB-DFP/ECFP4. The six matrices are shown in Additional file 1: Tables S4–S9. Table 2 summarizes the maximum, minimum, average and range of Tanimoto similarity values for each similarity matrix. By using the median similarity between ACC in the data sets, the ranges are the smallest for MACCS keys and ECFP4 (i.e., 0.51 and 0.49, respectively). Table 2 shows that the similarity matrices constructed using SB-DFP present a broader range of values (0.950 and 0.989) than those constructed using DFP (0.746 and 0.930).

**Table 2:** Range of Tanimoto similarity values in similarity matrices

Representation	MACCS keys (166-bit)				ECFP4 (2048-bit)			
	Minimum	Average	Maximum	Range	Minimum	Average	Maximum	Range
All compounds^a^	0.293	0.407	0.804	0.511	0.059	0.114	0.553	0.494
DFP	0.254	0.540	1.000	0.746	0.070	0.408	1.000	0.930
SB-DFP	0.050	0.342	1.000	0.950	0.011	0.185	1.000	0.989

^a^It should be noted that the comparisons involving the self-similarity of data sets does not reach a value of 1 and in some cases such self-similarity does not correspond to the highest value in the matrix row, that could be misinterpreted as the existence of pairs of databases more similar to each other than to themselves, which makes no sense. The matrices constructed by using DFP or SB-DFP do not present such problem, since when dealing with unique comparisons, a maximum of 1 is guaranteed for the diagonal of the matrix

The SB-DFP matrices also have lower average similarities between data sets than the DFP matrices (0.540 vs. 0.342 for MACCS keys and 0.408 vs. 0.185 for ECFP4, respectively). Based on these results, the representation that captures better the differences between data sets is SB-DFP/ECFP4. This result agrees with the relative “higher resolution” of SB-DFP/ECFP4 i.e., higher number of “1” bits discussed above (Table 1).

### SB-DFP to study inter-data set relationship

**Fig. 2:**
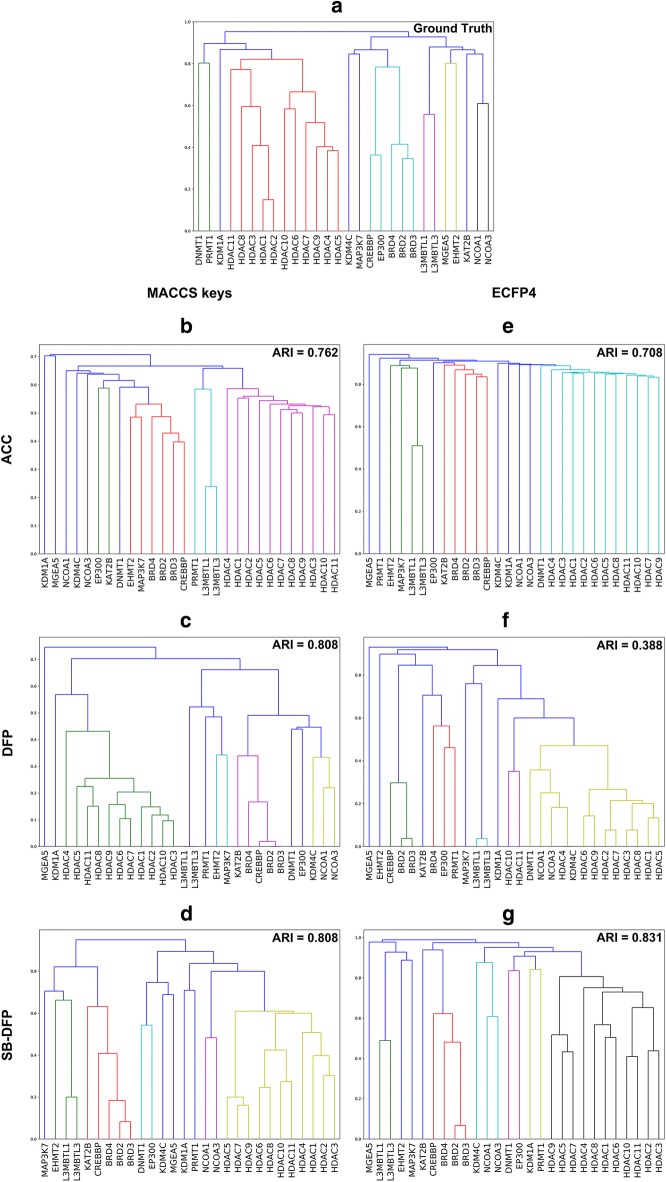
Dendograms for hierarchical clustering of targets computed with different approaches based in two molecular fingerprints, MACCS keys and ECFP4. **a** The ground truth; **b**, **e** all-compound comparisons (ACC); **c**, **f** database fingerprint (DFP); **d**, **g** statistical-based database fingerprint (SB-DFP). The Adjusted Rand Index (ARI) of each clustering is indicated in each panel. See main text for details

Figure 2 shows the dendrograms for each hierarchical clustering obtained with the corresponding distance matrices (vide supra). Analyzing the differences between data sets is not a trivial task and it is not straightforward evaluating the performance of a structural representation. In this work, we assessed the ability of the six representations listed above to recover the known relationships between epigenetic targets based on its sequence identity, using as metric the ARI at a level of 10 clusters and as ground truth the hierarchical clustering obtained from the distance form of the sequence identity matrix (vide supra). The level of ten clusters was selected as ground truth given its recovery of four groups of epigenetic targets with known relationships: group 1 containing BRDs 2–4, CREBBP and EP300; group 2 containing HDACs 1–11; group 3 including L3MBTLs 1 and 3; and group 4 consisting of NCOAs 1 and 3. According to the results, the best performing methods were those based on the SB-DFP, with ARI values of 0.831 for SB-DP/ECFP4 and 0.808 for SB-DFP/MACCS. Methods based on ACC had worst but similar performances for both fingerprints with ARI values of 0.762 and 0.708 for ACC/MACCS and ACC/ECFP4 respectively. Finally, methods based on DFP had contrasting performances, being DFP/MACCS tied as the second best method with an ARI value of 0.808 and DFP/ECFP4 the worst of them with an ARI value of 0.388.

### SB-DFP as template for similarity searching

**Fig. 3:**
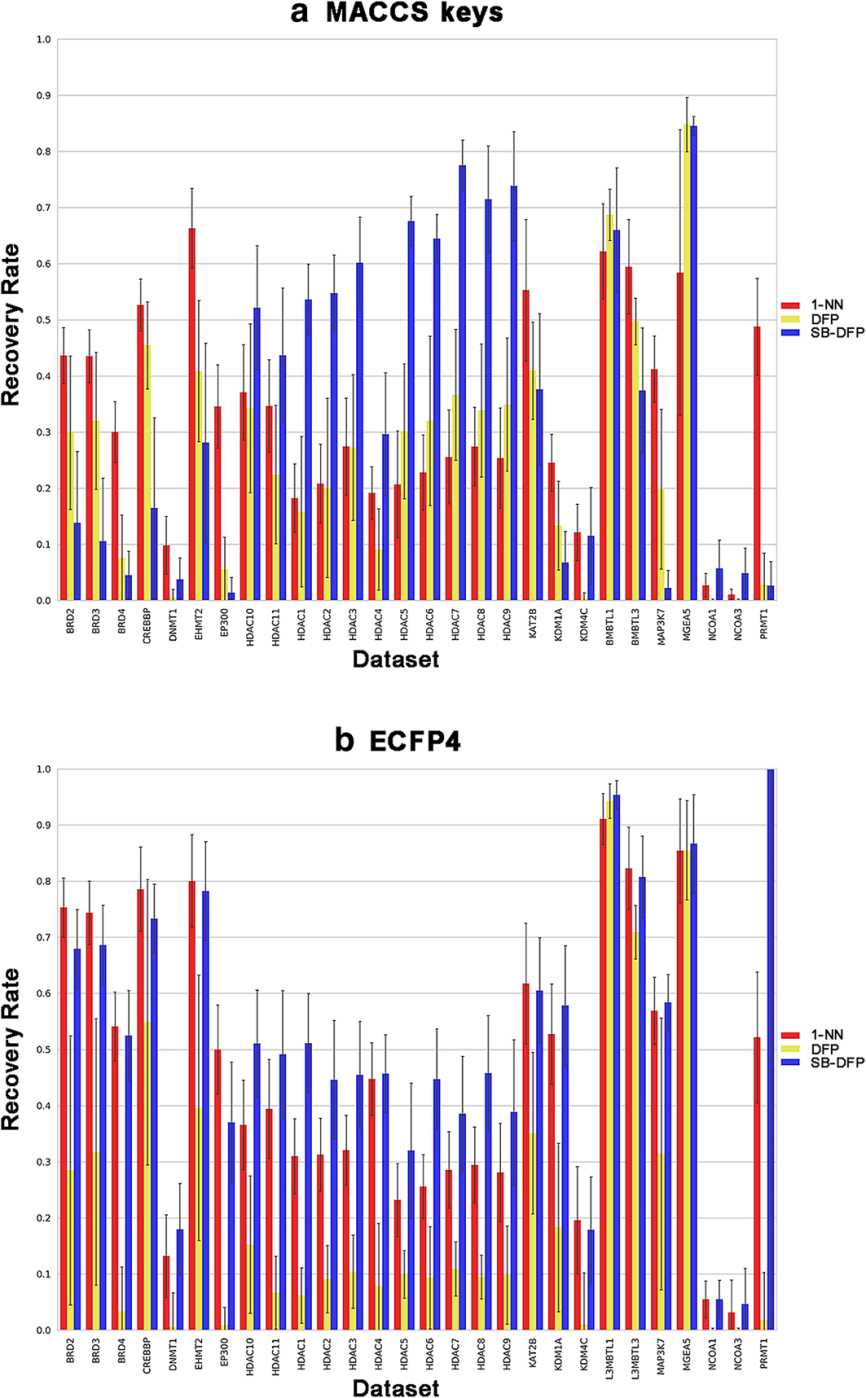
Early enrichment performance of similarity searches. Average recovery rates (selection set size equal to the number of ADCs) for three search strategies over 28 epigenetic data sets are reported in a histogram representation for **a** MACCS keys and **b** ECFP4. Standard deviations are displayed as error bars

**Fig. 4:**
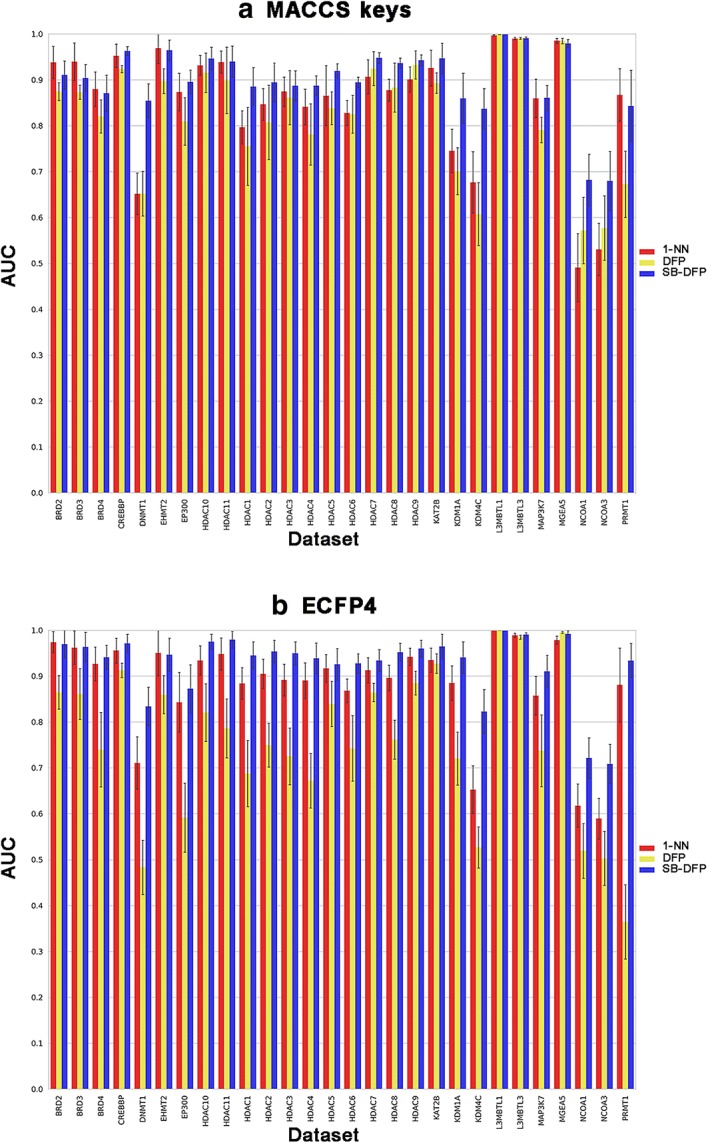
General performance of similarity searches. Average AUCs for three search strategies over 28 epigenetic data sets are reported in a histogram representation for **a** MACCS keys and **b** ECFP4. Standard deviations are displayed as error bars

All 28 epigenetic data sets were subjected to systematic fingerprint search calculations. To obtain statistically relevant data, from each data set, 100 compound reference sets of 10 compounds were randomly selected and used as query in six different representations: the fingerprints for each compound (1-NN), the DFP and the SB-DFP, the three of them based on ECFP4 and MACCS keys. For the six search strategies, Figs. 3 and 4 show the results of the RR and AUC, respectively. In terms of early enrichment, by using MACCS keys as molecular representation, the SB-DFP approach outperformed the other methods with an average RR of 35.3%, followed by 1-NN (33.1%) and DFP (26.4%). Similar trends were obtained using ECFP4, being the average RRs 50.2%, 46% and 21.5 for SB-DFP, 1-NN, and DFP respectively. Regarding to the global performance, the tendency was identical. The best performing method in both cases was SB-DFP, for MACCS keys with an average AUC of 0.898, followed by 1-NN and DFP with average AUCs of 0.853 and 0.824 respectively and for ECFP4 with average AUCs of 0.926, 0.882 and 0.755 for SB-DFP, 1-NN and DFP respectively. These results revealed the anticipated differences between high- and low-resolution fingerprints, since ECFP4 achieved higher RRs and AUCs for 1-NN searches, while for the single fingerprint searches the higher values corresponded to the most populated representations in terms of number of bits “1” (MACCS keys for DFP and ECFP4 for SB-DFP).

The results also illustrated the general data set-dependence of the similarity searching performance and the good success rates achieved for 2D fingerprint methods, since the best performing search strategy for each data set obtained an average RR of at least 50% in 22 of 28 cases, and an average AUC larger than 0.7 in all of them. By analyzing the individual performances, according to RRs (Table 3), SB-DFP was the best method for 17 cases, from which eight were based on MACCS keys, seven based on ECFP4 and two without significative difference between molecular fingerprints. The second best method was 1-NN with eight favorable cases by using ECFP4. For three data sets there was not significative difference between SB-DFP and 1-NN (Fig. 3). Additionally, the DFP representation was not the best performing method for any of the data sets studied.

**Table 3:** Average recovery rates

Dataset	MACCS keys (166-bit)			ECFP4 (2048-bit)		
	1-NN	DFP	SB-DFP	1-NN	DFP	SB-DFP
BRD2	43.7 (5.0)	29.9 (13.7)	13.8 (12.8)	**75.4 (5.2)**	28.4 (24.2)	68.0 (7.1)
BRD3	43.5 (4.8)	32.0 (12.3)	10.6 (11.3)	**74.4 (5.7)**	31.9 (23.8)	68.7 (7.1)
BRD4	30.0 (5.4)	7.6 (7.7)	4.5 (4.3)	**54.1 (6.2)**	2.7 (4.7)	52.6 (8.1)
CREBBP	52.7 (4.7)	45.5 (7.8)	16.5 (16.2)	**79.0 (5.4)**	55.6 (25.0)	73.7 (4.2)
DNMT1	9.9 (5.2)	0.5 (1.5)	3.8 (3.9)	12.9 (5.7)	0.0 (0.0)	**17.7 (7.1)**
EHMT2	66.3 (7.1)	40.9 (12.6)	28.1 (17.8)	**80.1 (8.0)**	40.2 (23.5)	78.4 (8.3)
EP300	34.6 (7.5)	5.5 (5.8)	1.4 (2.7)	**50.2 (7.7)**	0.7 (2.8)	37.0 (10.8)
HDAC10	37.1 (8.6)	34.2 (15.1)	**52.2 (11.1)**	36.5 (8.0)	15.4 (12.3)	**51.1 (9.5)**
HDAC11	34.7 (8.3)	22.5 (12.4)	43.7 (12.1)	39.6 (8.8)	6.6 (6.4)	**49.3 (11.3)**
HDAC1	18.2 (6.1)	15.8 (13.5)	**53.7 (6.3)**	30.9 (6.7)	6.3 (5.1)	51.1 (9.0)
HDAC2	20.9 (7.0)	20.1 (16.1)	**54.8 (6.9)**	31.3 (6.5)	9.1 (6.0)	44.7 (10.6)
HDAC3	27.5 (8.7)	27.3 (13.1)	**60.2 (8.1)**	32.0 (6.2)	10.4 (6.6)	45.4 (9.6)
HDAC4	19.2 (4.7)	9.1 (7.3)	29.6 (11.0)	**44.9 (6.2)**	7.9 (11.2)	**45.8 (7.0)**
HDAC5	20.7 (9.6)	30.2 (12.1)	**67.6 (4.4)**	23.1 (6.4)	10.0 (4.3)	32.0 (12.1)
HDAC6	22.8 (6.7)	32.0 (15.1)	**64.5 (4.3)**	25.7 (5.8)	9.3 (9.1)	44.6 (9.0)
HDAC7	25.6 (8.4)	36.6 (11.7)	**77.6 (4.5)**	28.4 (6.7)	11.0 (4.9)	38.6 (10.4)
HDAC8	27.4 (7.0)	33.9 (11.9)	**71.5 (9.5)**	29.6 (6.9)	9.5 (3.9)	46.2 (9.8)
HDAC9	25.4 (9.0)	34.9 (11.9)	**73.9 (9.7)**	27.7 (7.5)	9.6 (8.7)	38.4 (13.0)
KAT2B	55.3 (12.7)	41.0 (8.7)	37.6 (13.5)	**61.8 (10.8)**	35.3 (14.1)	**60.4 (9.4)**
KDM1A	24.6 (5.1)	13.3 (8.0)	6.8 (5.6)	53.3 (8.4)	18.3 (15.1)	**58.4 (10.0)**
KDM4C	12.2 (5.1)	0.4 (1.0)	11.5 (8.7)	**18.9 (6.4)**	0.1 (0.3)	17.1 (5.8)
L3MBTL1	62.2 (8.5)	68.8 (4.6)	66.0 (11.1)	91.1 (4.6)	94.5 (1.8)	**95.5 (2.3)**
L3MBTL3	59.5 (8.5)	49.7 (4.2)	37.4 (11.2)	**82.8 (6.6)**	71.1 (4.5)	81.1 (6.8)
MAP3K7	41.2 (6.0)	19.8 (14.3)	2.2 (3.1)	56.6 (5.2)	31.1 (23.8)	**58.0 (4.0)**
MGEA5	58.5 (25.6)	84.8 (4.9)	84.6 (1.7)	86.3 (3.5)	86.4 (2.0)	**87.6 (2.2)**
NCOA1	2.7 (2.1)	0.0 (0.2)	**5.7 (5.1)**	**5.5 (3.3)**	0.1 (0.3)	**5.5 (3.4)**
NCOA3	1.1 (0.9)	0.1 (0.2)	**4.9 (4.5)**	2.6 (1.4)	0.1 (0.3)	**4.1 (2.5)**
PRMT1	48.8 (8.7)	2.8 (5.7)	2.7 (4.3)	52.8 (10.5)	1.0 (3.8)	**55.3 (12.1)**
Average	**33.1 (19.6)**	**26.4 (22.8)**	**35.3 (29.1)**	**46.0 (25.9)**	**21.5 (28.4)**	**50.2 (23.8)**

The best performing methods for each dataset are shown in bold. If there were no significative difference between two or more methods, all of them are marked. Standard deviations are shown in parentheses

According to the AUCs values (Table 4), the best performing method for 23 data sets was SB-DFP, from which four were based on MACCS keys, 17 based on ECFP4 and two without significative difference between fingerprints. The overall second-best approach was 1-NN with better predictions for two data sets (one for each molecular fingerprint). In general, DFP had lower AUCs values as compared to the other two search methods (Table 4).

**Table 4:** Average areas under ROC curves

Dataset	MACCS keys (166-bit)			ECFP4		
	1-NN	DFP	SB-DFP	1-NN	DFP	SB-DFP
BRD2	0.938 (0.035)	0.875 (0.019)	0.911 (0.031)	**0.974 (0.023)**	0.865 (0.037)	0.970 (0.030)
BRD3	0.940 (0.041)	0.873 (0.015)	0.905 (0.029)	**0.962 (0.037)**	0.861 (0.056)	**0.964 (0.032)**
BRD4	0.880 (0.038)	0.821 (0.036)	0.871 (0.040)	0.927 (0.037)	0.740 (0.082)	**0.941 (0.026)**
CREBBP	0.953 (0.025)	0.924 (0.008)	0.963 (0.009)	0.956 (0.027)	0.913 (0.016)	**0.972 (0.020)**
DNMT1	0.652 (0.045)	0.652 (0.049)	**0.855 (0.037)**	0.711 (0.058)	0.484 (0.060)	0.834 (0.042)
EHMT2	**0.969 (0.033)**	0.897 (0.027)	0.965 (0.023)	0.951 (0.050)	0.860 (0.042)	0.947 (0.036)
EP300	0.874 (0.041)	0.810 (0.052)	**0.896 (0.026)**	0.843 (0.066)	0.592 (0.076)	0.873 (0.052)
HDAC10	0.932 (0.022)	0.916 (0.043)	0.946 (0.025)	0.934 (0.032)	0.821 (0.063)	**0.975 (0.016)**
HDAC11	0.939 (0.024)	0.899 (0.073)	0.940 (0.034)	0.948 (0.035)	0.786 (0.065)	**0.979 (0.018)**
HDAC1	0.797 (0.036)	0.755 (0.085)	0.886 (0.041)	0.884 (0.035)	0.688 (0.073)	**0.945 (0.030)**
HDAC2	0.847 (0.035)	0.808 (0.081)	0.895 (0.042)	0.905 (0.032)	0.750 (0.048)	**0.954 (0.024)**
HDAC3	0.875 (0.032)	0.862 (0.059)	0.888 (0.032)	0.892 (0.035)	0.725 (0.062)	**0.950 (0.025)**
HDAC4	0.841 (0.039)	0.781 (0.067)	0.888 (0.021)	0.890 (0.039)	0.672 (0.060)	**0.939 (0.034)**
HDAC5	0.866 (0.066)	0.838 (0.036)	**0.920 (0.016)**	0.917 (0.030)	0.840 (0.049)	**0.926 (0.035)**
HDAC6	0.828 (0.028)	0.825 (0.042)	0.895 (0.011)	0.868 (0.026)	0.743 (0.072)	**0.928 (0.021)**
HDAC7	0.907 (0.037)	0.925 (0.037)	**0.948 (0.012)**	0.913 (0.027)	0.864 (0.020)	0.934 (0.024)
HDAC8	0.878 (0.024)	0.883 (0.054)	0.937 (0.011)	0.896 (0.028)	0.762 (0.043)	**0.953 (0.019)**
HDAC9	0.901 (0.028)	0.933 (0.031)	0.943 (0.012)	0.942 (0.019)	0.885 (0.026)	**0.960 (0.018)**
KAT2B	0.926 (0.039)	0.893 (0.022)	0.947 (0.033)	0.935 (0.027)	0.928 (0.022)	**0.965 (0.027)**
KDM1A	0.745 (0.048)	0.701 (0.051)	0.860 (0.055)	0.885 (0.038)	0.721 (0.058)	**0.941 (0.034)**
KDM4C	0.677 (0.067)	0.608 (0.069)	**0.837 (0.044)**	0.653 (0.052)	0.527 (0.045)	0.823 (0.048)
L3MBTL1	0.997 (0.001)	0.999 (0.000)	**1.000 (0.000)**	1.000 (0.000)	1.000 (0.000)	**1.000 (0.000)**
L3MBTL3	0.990 (0.003)	**0.991 (0.002)**	**0.991 (0.003)**	0.989 (0.005)	0.985 (0.004)	**0.990 (0.005)**
MAP3K7	0.860 (0.042)	0.791 (0.028)	0.861 (0.027)	0.858 (0.042)	0.738 (0.079)	**0.911 (0.035)**
MGEA5	0.985 (0.005)	0.985 (0.006)	0.979 (0.009)	0.979 (0.009)	**0.996 (0.002)**	0.992 (0.007)
NCOA1	0.491 (0.074)	0.572 (0.073)	0.682 (0.056)	0.618 (0.047)	0.519 (0.060)	**0.722 (0.044)**
NCOA3	0.530 (0.057)	0.577 (0.071)	0.680 (0.064)	0.590 (0.045)	0.503 (0.059)	**0.709 (0.043)**
PRMT1	0.867 (0.058)	0.673 (0.072)	0.843 (0.078)	0.881 (0.081)	0.365 (0.081)	**0.934 (0.037)**
Average	**0.853 (0.132)**	**0.824 (0.129)**	**0.898 (0.082)**	**0.882 (0.113)**	**0.755 (0.171)**	**0.926 (0.077)**

The best performing methods for each dataset are shown in bold. If there were no significative difference between two or more methods, all of them are marked. Standard deviations are shown in parentheses

Remarkably, the search method based on SB-DFP could be applied in at least 20 out of the 28 data sets studied leading to the best RRs, with the additional advantage over 1-NN that the speed of calculation is *N* times faster (with *N* being the number of compounds used as query). This fact is because the number of comparisons needed for the screening is always equal to the number of compounds in the screened database in contrast to 1-NN, where this number scale with the number of compounds used as query.

## Conclusions and perspectives

Here we presented the statistical-based database fingerprint (SB-DFP) as a novel general approach to generate single fingerprints of compound databases based on binomial proportion comparisons. In this work we shown its implementation for two molecular fingerprints (e.g., ECFP4 and MACCS keys) and one specific reference set (e.g., ZINC). However, the applicability of SB-DFP can be extended to any binary fingerprint and to other reference sets. Using as a case study a recently published set of 28 epigenetic compound sets with therapeutic relevance, we illustrate the application of SB-DFP to capture the inter-data sets relationships and to perform similarity searching. For the data sets explored in this work the largest set has 2740 compounds (as deposited in ChEMBL) but SB-DFP could be applied to other larger compound data with relevance in drug or probe discovery. Despite the fact that no quantitative analysis was performed in terms of speed of calculation, it is clear that single fingerprint approaches to represent compound databases are faster because they depend on single rather than multiple comparisons.

Two major perspectives of the SB-DFP approach are application in high throughput virtual screening and target identification. To these ends, studies involving different molecular fingerprints, target-associated compound sets and reference data sets would be required, as well as exhaustive validations of their performance. Part of this work in ongoing and will be reported in due course.

## Additional file

**Additional file 1: Table S1.** Average similarity searching performances for SB-DFP constructed at different confidence levels. **Table S2.** “1” bits count for 15,403,690 compounds taken from ZINC using MACCS keys. **Table S3.** “1” bits count for 15,403,690 compounds taken from ZINC using ECFP4. **Table S4.** Similarity matrix of compound data sets computed as the median Tanimoto coefficient between its compounds using MACCS keys. **Table S5.** Similarity matrix of compound data sets computed as Tanimoto coefficient between its DFP based on MACCS keys. **Table S6.** Similarity matrix of compound data sets computed as Tanimoto coefficient between its SB-DFP based on MACCS keys. **Table S7.** Similarity matrix of compound data sets computed as the median Tanimoto coefficient between its compounds using ECFP4. **Table S8.** Similarity matrix of compound data sets computed as Tanimoto coefficient between its DFP based on ECFP4. **Table S9.** Similarity matrix of compound data sets computed as Tanimoto coefficient between its SB-DFP based on ECFP4. **Table S10.** Sequence identity matrix of targets computed from Clustal Omega alignments.

## Abbreviations

ACC: all compound comparisons
ADC: active database of compounds
ARI: Adjusted Rand Index
AUC: area under the curve
DFP: database fingerprint
ECFP4: extended connectivity fingerprint of diameter four
k-NN: k-nearest neighbors
MACCS: molecular access system
ROC: receiver operating characteristic
RR: recovery rate
SB-DFP: statistical-based database fingerprint
UniProt: Universal Protein Knowledgebase
