# Supplementary material for: Statistical-based database fingerprint: chemical space dependent representation of compound databases
Source: J Cheminform. 2018 Nov 22;10:55. doi: 10.1186/s13321-018-0311-x (PMC6755589; doi:10.1186/s13321-018-0311-x)
Supplement: Supplementary file 1 — Additional file 1: Table S1. Average similarity searching performances for SB-DFP constructed at different confidence levels. Table S2. “1” bits count for 15,403,690 compounds taken from ZINC using MACCS keys. Table S3. “1” bits count for 15,403,690 compounds taken from ZINC using ECFP4. Table S4. Similarity matrix of compound data sets computed as the median Tanimoto coefficient between its compounds using MACCS keys. Table S5. Similarity matrix of compound data sets computed as Tanimoto coefficient between its DFP based on MACCS keys. Table S6. Similarity matrix of compound data sets computed as Tanimoto coefficient between its SB-DFP based on MACCS keys. Table S7. Similarity matrix of compound data sets computed as the median Tanimoto coefficient between its compounds using ECFP4. Table S8. Similarity matrix of compound data sets computed as Tanimoto coefficient between its DFP based on ECFP4. Table S9. Similarity matrix of compound data sets computed as Tanimoto coefficient between its SB-DFP based on ECFP4. Table S10. Sequence identity matrix of targets computed from Clustal Omega alignments. [file 13321_2018_311_MOESM1_ESM.pdf]

Supporting Information

Statistical-Based Database Fingerprint: Chemical space dependent representation of compound databases

Norberto Sánchez-Cruz, José L. Medina-Franco

Contents Page

**Table S1**	Average similarity searching performances for SB-DFP constructed at different confidence levels.	S2
**Table S2**	“1” bits count for 15,403,690 compounds taken from ZINC using MACCS keys.	S3
**Table S3**	“1” bits count for 15,403,690 compounds taken from ZINC using ECFP4.	S4
**Table S4**	Similarity matrix of compound data sets computed as the median Tanimoto coefficient between its compounds using MACCS keys.	S6
**Table S5**	Similarity matrix of compound data sets computed as Tanimoto coefficient between its DFP based on MACCS keys.	S7
**Table S6**	Similarity matrix of compound data sets computed as Tanimoto coefficient between its SB-DFP based on MACCS keys.	S8
**Table S7**	Similarity matrix of compound data sets computed as the median Tanimoto coefficient between its compounds using ECFP4.	S9
**Table S8**	Similarity matrix of compound data sets computed as Tanimoto coefficient between its DFP based on ECFP4.	S10
**Table S9**	Similarity matrix of compound data sets computed as Tanimoto coefficient between its SB-DFP based on ECFP4.	S11
**Table S10**	Sequence identity matrix of targets computed from Clustal Omega alignments.	S12

Table S1. Average similarity searching performances for SB-DFP constructed at different confidence levels. [a]

Confidence level (p-value)	ECFP4		MACCS keys (166-bit)	
	Average RR	Average AUC	Average RR	Average AUC
**99% (p<0.01)**	50.2 (23.8)	**0.926 (0.077)**	**35.3 (29.1)**	**0.897 (0.081)**
**99.9% (p<0.001)**	**50.8 (23.1)**	0.924 (0.080)	29.4 (31.1)	0.886 (0.090)
**99.99% (p<0.0001)**	50.7 (22.9)	0.918 (0.083)	28.4 (31.5)	0.872 (0.095)
**99.999% (p>0.00001)**	49.9 (22.9)	0.910 (0.088)	28.1 (31.6)	0.835 (0.117)
**99,9999% (p>0.000001)**	49.1 (22.9)	0.904 (0.095)	28.0 (31.2)	0.775 (0.152)

[a] The best performing methods for each metric is shown in bold. Standard deviations are shown in parentheses.

Table S2. “1” bits count for 15,403,690 compounds taken from ZINC using MACCS keys. [a]

Bit	Count	Bit	Count
1	0	84	2223311
2	0	85	8756111
3	0	86	7163984
4	0	87	4705749
5	0	88	4850760
6	0	89	1733047
7	0	90	6989685
8	68484	91	7298770
9	0	92	9776977
10	0	93	5830136
11	252951	94	5038471
12	0	95	7395965
13	2577	96	9582355
14	1	97	7148408
15	1139	98	7818322
16	51	99	737232
17	120645	100	10892579
18	0	101	5066065
19	539512	102	2305464
20	0	103	2184371
21	13801	104	7672347
22	980413	105	5276043
23	266605	106	5808622
24	2271	107	5346980
25	481478	108	5733218
26	69481	109	4973082
27	49797	110	11198117
28	195235	111	11451517
29	37	112	4142913
30	8	113	5543483
31	78	114	3776147
32	1414636	115	4968917
33	1458080	116	5321466
34	229666	117	11740905
35	0	118	9753376
36	2645901	119	298365
37	2075227	120	9789376
38	3132381	121	11735266
39	17230	122	10327864
40	17705	123	2394576
41	674850	124	5256885
42	2956810	125	10178681
43	1106726	126	5931192
44	0	127	6368134
45	108153	128	8543630
46	711023	129	8937760
47	2397840	130	2259218
48	17238	131	5681306
49	4776081	132	7767731
50	302418	133	7972604
51	1615902	134	5348961
52	3172149	135	6763521
53	1584443	136	5787409
54	1902517	137	12882269
55	1604604	138	8842064
56	5	139	1764188
57	3927104	140	2497776
58	1605405	141	4788569
59	2541833	142	12912635
60	1659158	143	6368134
61	1659219	144	9179678
62	4919105	145	9677648
63	1	146	6318016
64	2556961	147	10077906
65	8340196	148	10837836
66	2065239	149	8844114
67	1717565	150	9598365
68	821	151	12232870
69	1029978	152	7554412
70	368744	153	12732204
71	704219	154	11833635
72	2389368	155	12011232
73	1659232	156	14699565
74	2899042	157	8868254
75	8190584	158	14647306
76	326627	159	10997462
77	6371679	160	12926448
78	297886	161	15101534
79	7611630	162	14444086
80	7637694	163	14357547
81	4624987	164	14330953
82	6575993	165	15312239
83	9046831	166	0

[a] MACCS keys 166-bit

Table S3. “1” bits count for 15,403,690 compounds taken from ZINC using ECFP4. [a]

Bit	Count	Bit	Count	Bit	Count	Bit	Count	Bit	Count	Bit	Count	Bit	Count	Bit	Count	Bit	Count	Bit	Count	Bit	Count	Bit	Count	Bit	Count	Bit	Count	Bit	Count	Bit	Count
1	28098	65	69913	129	106199	193	349473	257	38459	321	44331	411	200231	449	621817	513	138406	577	88503	641	175267	705	88761	769	190050	833	347914	897	750663	961	230179
2	5509918	66	99913	130	55821	194	766250	258	792613	322	88445	412	159988	450	99377	514	44290	578	76335	642	176497	706	55005	770	47052	834	436619	898	101671	962	246012
3	819496	67	129372	131	282022	195	91185	259	80487	323	2262337	413	223822	451	67231	515	161167	579	27363	643	75224	707	97045	771	43730	835	213901	899	310947	963	40365
4	116916	68	191462	132	122054	196	183102	260	107023	324	287538	414	294674	452	226214	516	148853	580	75098	644	59429	708	60549	772	183180	836	380117	900	199293	964	51098
5	196011	69	137781	133	37877	197	250631	261	59559	325	68960	415	29090	453	44358	517	35756	581	85553	645	50295	709	289439	773	197739	837	67361	901	140064	965	159307
6	222420	70	361181	134	95354	198	2844716	262	112113	326	522745	416	55828	454	26183	518	124186	582	308987	646	138505	710	36784	774	65262	838	167790	902	564863	966	124130
7	102737	71	59946	135	59682	199	139738	263	154007	327	64917	417	33856	455	112836	519	101284	583	108316	647	46502	711	146843	775	138634	839	60678	903	59141	967	99060
8	72883	72	56545	136	68434	200	92018	264	293988	328	164481	418	87015	456	235689	520	76526	584	61144	648	133647	712	82267	776	46531	840	77349	904	40930	968	202039
9	636553	73	69021	137	77273	201	26564	265	87836	329	151721	419	41314	457	156222	521	59063	585	114142	649	82989	713	50866	777	46823	841	163783	905	48939	969	134416
10	231405	74	58562	138	42358	202	125741	266	400667	330	92635	420	337926	458	164064	522	94806	586	610720	650	131647	714	140621	778	106433	842	3157097	906	46357	970	44393
11	61884	75	675284	139	89930	203	188315	267	43894	331	113033	421	68287	459	128842	523	114645	587	424064	651	12406687	715	67041	779	83714	843	81422	907	266216	971	113541
12	233619	76	179341	140	137012	204	238124	268	120378	332	38598	422	164835	460	173000	524	699257	588	244669	652	156808	716	1363612	780	126476	844	1338342	908	92016	972	164294
13	59863	77	137861	141	689194	205	219578	269	196097	333	130518	423	200334	461	251683	525	170484	589	142769	653	514052	717	471454	781	67460	845	123895	909	168410	973	31501
14	1170965	78	169666	142	55573	206	74410	270	32064	334	434270	424	59502	462	305330	526	150584	590	91018	654	116327	718	43687	782	1186623	846	158312	910	418947	974	162343
15	955803	79	115115	143	141829	207	261169	271	144986	335	468153	425	56785	463	78106	527	540548	591	142693	655	198164	719	1552261	783	127325	847	109064	911	148412	975	1264879
16	55056	80	491170	144	182066	208	176781	272	126349	336	341031	426	474437	464	201429	528	316502	592	390828	656	33316	720	57999	784	297643	848	298233	912	236798	976	74674
17	45255	81	12031271	145	39567	209	59092	273	119004	337	36094	427	143059	465	698724	529	96309	593	323731	657	4238350	721	220365	785	223942	849	94308	913	119347	977	136027
18	263888	82	156831	146	885789	210	300664	274	55939	338	82474	428	159910	466	86694	530	45018	594	49274	658	59669	722	169555	786	443820	850	435869	914	243244	978	217490
19	186429	83	71346	147	199497	211	116184	275	43173	339	156540	429	308523	467	93889	531	199696	595	169189	659	299342	723	148018	787	91762	851	145824	915	50449	979	533341
20	47931	84	141439	148	864299	212	81308	276	108464	340	173583	430	107127	468	83443	532	349373	596	87007	660	95588	724	139011	788	721146	852	32226	916	273574	980	109472
21	63634	85	231346	149	62317	213	100473	277	124097	341	36551	431	82306	469	113688	533	164969	597	194631	661	75797	725	696984	789	78764	853	72904	917	115874	981	631599
22	89647	86	95071	150	151177	214	295601	278	208988	342	851848	432	95192	470	203235	534	88152	598	284172	662	120164	726	189270	790	82360	854	114343	918	467009	982	35409
23	46210	87	40126	151	79084	215	109272	279	145459	343	69116	433	71536	471	82648	535	210110	599	420230	663	79727	727	67681	791	752534	855	123151	919	101281	983	41893
24	60082	88	146242	152	146035	216	113899	280	95164	344	150699	434	101457	472	276836	536	103823	600	192799	664	49500	728	86546	792	36251	856	425236	920	85527	984	112468
25	84970	89	70928	153	89956	217	1476455	281	162843	345	132051	435	43860	473	87384	537	168453	601	212773	665	449336	729	757381	793	31472	857	80907	921	109083	985	350126
26	84479	90	103096	154	45594	218	114186	282	347805	346	163205	436	44626	474	425929	538	60943	602	64397	666	133914	730	87335	794	27563	858	226884	922	87654	986	107324
27	54594	91	796064	155	88333	219	509657	283	111938	347	23461	437	23560	475	439276	539	25808	603	156238	667	333578	731	152349	795	1035349	859	63114	923	112946	987	35960
28	115973	92	89013	156	157319	220	154445	284	3807442	348	110735	438	335233	476	119335	540	166554	604	138194	668	1365661	732	90512	796	118275	860	115340	924	48944	988	228473
29	53572	93	689850	157	50252	221	160716	285	64793	349	143028	439	55980	477	103887	541	120806	605	86364	669	43669	733	205838	797	94625	861	94403	925	39745	989	55790
30	847853	94	229945	158	284216	222	171104	286	100174	350	104811	440	40202	478	87757	542	149401	606	79112	670	37750	734	98810	798	265153	862	121936	926	149891	990	194687
31	162057	95	914421	159	409394	223	680234	287	108760	351	1615310	441	279391	479	127338	543	52018	607	623449	671	39734	735	297960	799	587716	863	187006	927	9189665	991	57868
32	39235	96	138979	160	51306	224	109708	288	174560	352	130798	442	594613	480	62298	544	132236	608	301359	672	64551	736	199275	800	130652	864	116530	928	194845	992	60180
33	121459	97	39781	161	156593	225	67303	289	184309	353	604063	443	119725	481	62293	545	106286	609	65928	673	56079	737	710732	801	576835	865	55325	929	36931	993	116726
34	196899	98	73659	162	103494	226	57686	290	616295	354	86323	444	57086	482	210351	546	81312	610	243553	674	47761	738	80383	802	52642	866	86882	930	99215	994	89895
35	351292	99	122290	163	1298661	227	195369	291	203090	355	278489	445	1094224	483	183113	547	84206	611	146967	675	853272	739	321818	803	1342006	867	90258	931	119977	995	233862
36	106336	100	192557	164	60406	228	662753	292	222456	356	109354	446	120659	484	75873	548	80958	612	167955	676	2710769	740	1972724	804	148318	868	109848	932	122467	996	54530
37	78062	101	59718	165	71547	229	24546	293	67329	357	65051	447	757399	485	339898	549	217497	613	59557	677	141744	741	45506	805	147887	869	56364	933	758814	997	186119
38	138810	102	79452	166	619311	230	37686	294	117220	358	206092	448	53008	486	166408	550	92054	614	141430	678	188278	742	82156	806	163818	870	83949	934	85412	998	194421
39	194102	103	771599	167	117337	231	288925	295	3866630	359	135375	411	200231	487	2561482	551	110437	615	94195	679	151119	743	68764	807	440620	871	169748	935	120135	999	36476
40	111687	104	49481	168	190546	232	999497	296	242439	360	54548	412	159988	488	103810	552	93988	616	109910	680	361313	744	149433	808	11947839	872	71016	936	7913458	1000	175377
41	55295	105	234539	169	173240	233	58219	297	92464	361	129645	413	223822	489	194420	553	923876	617	34281	681	408780	745	183055	809	77955	873	113561	937	101388	1001	132428
42	2254111	106	153342	170	84969	234	560437	298	104433	362	760144	414	294674	490	352870	554	50944	618	125379	682	106306	746	728239	810	635821	874	72864	938	125903	1002	53048
43	468791	107	71708	171	81377	235	68300	299	132623	363	241943	415	29090	491	459682	555	283633	619	180623	683	96574	747	49149	811	43376	875	42857	939	61461	1003	64507
44	776107	108	256197	172	80347	236	997675	300	132654	364	182706	416	55828	492	266498	556	236970	620	114730	684	39987	748	849592	812	301446	876	6910244	940	69817	1004	58005
45	26883	109	220612	173	216238	237	101670	301	82487	365	105395	417	33856	493	65195	557	343523	621	28064	685	158683	749	53805	813	91984	877	141547	941	322952	1005	286483
46	587270	110	69201	174	57985	238	867581	302	240703	366	138928	418	87015	494	145598	558	207621	622	135235	686	100163	750	158798	814	291446	878	62161	942	131800	1006	643241
47	160663	111	144020	175	25559	239	128069	303	160195	367	1263151	419	41314	495	93866	559	93944	623	210073	687	83220	751	403829	815	119223	879	207386	943	443911	1007	103763
48	29915	112	51210	176	58495	240	124578	304	88236	368	41695	420	337926	496	68878	560	181666	624	199171	688	236682	752	199197	816	246921	880	203279	944	61396	1008	152663
49	185682	113	147861	177	37046	241	148404	305	48627	369	201087	421	68287	497	99990	561	155504	625	188894	689	132123	753	73919	817	1180743	881	80373	945	107955	1009	155104
50	61099	114	100857	178	131718	242	525696	306	378477	370	519927	422	164835	498	297711	562	2261633	626	48180	690	871164	754	112275	818	54785	882	2162471	946	120797	1010	833971
51	184900	115	1731543	179	66007	243	723918	307	49542	371	63843	423	200334	499	20095	563	732001	627	184457	691	207268	755	172569	819	90254	883	62022	947	65156	1011	49938
52	104361	116	165622	180	51929	244	69662	308	79392	372	421495	424	59502	500	117998	564	85682	628	27137	692	73175	756	187612	820	285264	884	484599	948	334518	1012	144026
53	100222	117	1214160	181	156369	245	102553	309	145443	373	141097	425	56785	501	113894	565	347865	629	70052	693	343207	757	123282	821	277421	885	93457	949	118621	1013	67813
54	99737	118	1422792	182	33015	246	297090	310	211023	374	416979	426	474437	502	333972	566	71424	630	161438	694	53488	758	261864	822	312614	886	401667	950	251829	1014	703219
55	238269	119	844634	183	61468	247	251540	311	1733839	375	239235	427	143059	503	564231	567	41285	631	58221	695	421928	759	143805	823	48309	887	124419	951	1582939	1015	165187
56	64821	120	731759	184	34398	248	113545	312	179370	376	48318	428	159910	504	227900	568	213110	632	53376	696	6068107	760	295910	824	170874	888	109994	952	118785	1016	87056
57	96048	121	102501	185	964188	249	1012156	313	246495	377	40394	429	308523	505	624828	569	75502	633	132002	697	59292	761	327506	825	63764	889	319924	953	132025	1017	26400
58	321876	122	257458	186	56323	250	1259551	314	184436	378	152318	430	107127	506	52736	570	169392	634	55841	698	103946	762	46745	826	77614	890	35876	954	94235	1018	689101
59	193591	123	115723	187	326044	251	194358	315	2468063	379	7507884	431	82306	507	181838	571	129964	635	103842	699	104732	763	229660	827	76923	891	1046558	955	113193	1019	163898
60	113167	124	122022	188	83116	252	120289	316	125101	380	97394	432	95192	508	791605	572	94035	636	187256	700	2258570	764	157092	828	128805	892	280665	956	96861	1020	7139008
61	71309	125	37396	189	57263	253	392707	317	146184	381	214494	433	71536	509	55995	573	45600	637	69963	701	46843	765	88994	829	369193	893	320675	957	36682	1021	178896
62	96120	126	153943	190	79349	254	78962	318	296296	382	254315	434	101457	510	48453	574	942233	638	81389	702	292272	766	143467	830	59155	894	153498	958	76838	1022	296768
63	287977	127	100917	191	101047	255	127446	319	342294	383	80976	435	43860	511	157816	575	51464	639	307045	703	142074	767	222795	831	51598	895	93062	959	65431	1023	103150
64	105297	128	120053	192	2076094	256	1324738	320	912267	384	781674	436	44626	512	190083	576	279895	640	26454	704	652885	768	107420	832	540199	896	166913	960	113047	1024	59603

Table S3. *(cont)*

Bit	Count	Bit	Count	Bit	Count	Bit	Count	Bit	Count	Bit	Count	Bit	Count	Bit	Count	Bit	Count	Bit	Count	Bit	Count	Bit	Count	Bit	Count	Bit	Count	Bit	Count	Bit	Count
1025	105234	1089	8338360	1153	8852968	1217	186434	1281	115039	1345	125890	1409	60552	1473	135988	1537	2342706	1601	30096	1665	241496	1729	65785	1793	145938	1857	260313	1921	573166	1985	420545
1026	246449	1090	404098	1154	173178	1218	55325	1282	38684	1346	45338	1410	252896	1474	162836	1538	77794	1602	154775	1666	698877	1730	1686282	1794	90578	1858	30968	1922	247486	1986	635006
1027	89608	1091	98043	1155	256806	1219	189582	1283	170065	1347	40582	1411	1742839	1475	55941	1539	55931	1603	413765	1667	102561	1731	93229	1795	122009	1859	185586	1923	255344	1987	123258
1028	735774	1092	182903	1156	305065	1220	84536	1284	180591	1348	63342	1412	116371	1476	121585	1540	262098	1604	765912	1668	92850	1732	496048	1796	372690	1860	464158	1924	323641	1988	43557
1029	4017419	1093	58857	1157	61846	1221	79266	1285	208404	1349	184825	1413	70687	1477	1691432	1541	138496	1605	1121724	1669	91942	1733	22977	1797	110878	1861	433399	1925	149321	1989	96876
1030	68802	1094	107434	1158	358199	1222	531948	1286	469292	1350	1425858	1414	92871	1478	95289	1542	82531	1606	154973	1670	206124	1734	214431	1798	49604	1862	207556	1926	53223	1990	933120
1031	79917	1095	48670	1159	177920	1223	84959	1287	71772	1351	85905	1415	115202	1479	45567	1543	783826	1607	57121	1671	1303567	1735	237187	1799	162476	1863	137432	1927	614944	1991	411830
1032	120087	1096	118583	1160	163512	1224	78738	1288	57064	1352	196763	1416	120048	1480	131477	1544	53624	1608	378120	1672	260067	1736	38550	1800	521450	1864	191887	1928	83791	1992	1257840
1033	483572	1097	428879	1161	1313593	1225	1318146	1289	112510	1353	60824	1417	494779	1481	4114609	1545	599090	1609	116303	1673	19094	1737	69301	1801	294250	1865	101169	1929	3015129	1993	363855
1034	114239	1098	952931	1162	84236	1226	108052	1290	107398	1354	98139	1418	397092	1482	295337	1546	102900	1610	103360	1674	137566	1738	35504	1802	70705	1866	77678	1930	363364	1994	352480
1035	463472	1099	31720	1163	1016194	1227	706462	1291	337141	1355	65499	1419	60760	1483	220171	1547	63778	1611	46921	1675	37381	1739	1175147	1803	91764	1867	1867121	1931	134091	1995	60804
1036	381929	1100	264205	1164	1202308	1228	104146	1292	107359	1356	91425	1420	34898	1484	84236	1548	60515	1612	137939	1676	65247	1740	102106	1804	355107	1868	55884	1932	61946	1996	56943
1037	98859	1101	124688	1165	1237197	1229	120389	1293	751913	1357	102126	1421	140088	1485	49122	1549	126361	1613	113232	1677	68549	1741	91317	1805	126994	1869	122521	1933	121491	1997	162195
1038	27511	1102	838213	1166	40893	1230	350959	1294	58932	1358	1145824	1422	36114	1486	94546	1550	87093	1614	70668	1678	77138	1742	245205	1806	92568	1870	43286	1934	43099	1998	137855
1039	73854	1103	172425	1167	91123	1231	62608	1295	279470	1359	64907	1423	184409	1487	94272	1551	60578	1615	192300	1679	114014	1743	238227	1807	75971	1871	92526	1935	492521	1999	586450
1040	2687463	1104	80552	1168	115103	1232	188335	1296	711414	1360	204270	1424	250380	1488	772995	1552	63934	1616	57181	1680	158042	1744	63055	1808	52584	1872	96989	1936	92610	2000	479185
1041	82085	1105	202396	1169	172783	1233	191897	1297	85851	1361	120218	1425	152923	1489	71314	1553	165740	1617	88477	1681	111004	1745	42687	1809	394045	1873	113828	1937	88885	2001	649523
1042	182268	1106	304186	1170	98708	1234	586540	1298	114006	1362	59633	1426	59663	1490	94822	1554	76483	1618	159023	1682	159216	1746	592069	1810	80704	1874	14146020	1938	196363	2002	50558
1043	92396	1107	118970	1171	212225	1235	55303	1299	40487	1363	432049	1427	70695	1491	111548	1555	483745	1619	136410	1683	257390	1747	103764	1811	391198	1875	27740	1939	79589	2003	148661
1044	558294	1108	83261	1172	1037255	1236	181430	1300	98838	1364	177577	1428	310419	1492	75770	1556	85766	1620	69081	1684	2515254	1748	180663	1812	82394	1876	79070	1940	233173	2004	51544
1045	37570	1109	69453	1173	59080	1237	864064	1301	223670	1365	59917	1429	65915	1493	73971	1557	90365	1621	127451	1685	87003	1749	44607	1813	110011	1877	438565	1941	74391	2005	883970
1046	345253	1110	85415	1174	143068	1238	163748	1302	50197	1366	1430389	1430	88604	1494	26477	1558	52215	1622	44986	1686	365934	1750	157982	1814	151242	1878	125062	1942	111653	2006	42228
1047	57269	1111	289514	1175	101681	1239	1354141	1303	115771	1367	290558	1431	415184	1495	76887	1559	21398	1623	75691	1687	69678	1751	12893880	1815	41209	1879	65126	1943	56404	2007	112646
1048	275216	1112	69394	1176	115737	1240	86763	1304	79362	1368	185609	1432	47130	1496	64101	1560	42597	1624	148067	1688	165000	1752	77364	1816	88751	1880	265676	1944	212809	2008	178478
1049	211718	1113	75202	1177	52587	1241	93649	1305	99015	1369	104056	1433	57787	1497	162285	1561	88155	1625	208374	1689	73950	1753	125898	1817	3429899	1881	58872	1945	57212	2009	77366
1050	444606	1114	37044	1178	42500	1242	65463	1306	70902	1370	172275	1434	81518	1498	60707	1562	146137	1626	80151	1690	217633	1754	83369	1818	236922	1882	62322	1946	123630	2010	1932595
1051	242082	1115	1726177	1179	44929	1243	67650	1307	57315	1371	143758	1435	209808	1499	78408	1563	200991	1627	470862	1691	1672815	1755	3059930	1819	111083	1883	96389	1947	368900	2011	292646
1052	126811	1116	129466	1180	106522	1244	1065240	1308	58634	1372	46074	1436	162789	1500	177220	1564	138998	1628	581831	1692	531083	1756	70434	1820	254682	1884	248971	1948	22090	2012	47763
1053	30149	1117	33514	1181	401046	1245	105791	1309	167056	1373	94547	1437	82488	1501	23137	1565	1000789	1629	57401	1693	202029	1757	164288	1821	218858	1885	254646	1949	542638	2013	193502
1054	49531	1118	83727	1182	103841	1246	351668	1310	1272567	1374	281882	1438	143385	1502	347396	1566	69306	1630	71787	1694	352285	1758	105706	1822	46207	1886	77227	1950	136097	2014	195242
1055	89629	1119	375259	1183	307233	1247	60087	1311	165389	1375	172192	1439	107754	1503	100879	1567	47398	1631	122974	1695	55609	1759	225956	1823	171409	1887	84736	1951	80939	2015	252424
1056	290615	1120	80191	1184	895317	1248	54422	1312	111152	1376	157394	1440	86304	1504	126756	1568	1214095	1632	775518	1696	247862	1760	180405	1824	1360507	1888	289321	1952	596823	2016	48827
1057	279914	1121	168347	1185	283405	1249	53238	1313	752629	1377	46168	1441	582737	1505	868655	1569	237294	1633	102918	1697	865827	1761	189158	1825	178217	1889	201539	1953	128889	2017	102615
1058	12938218	1122	400193	1186	64781	1250	259132	1314	199588	1378	97501	1442	482623	1506	81251	1570	107983	1634	134736	1698	1198525	1762	153066	1826	1012451	1890	63199	1954	223315	2018	183658
1059	148182	1123	88144	1187	83404	1251	53945	1315	57116	1379	40141	1443	272471	1507	487269	1571	230762	1635	748689	1699	60773	1763	312716	1827	67214	1891	29289	1955	164181	2019	378982
1060	37344	1124	223748	1188	231697	1252	79677	1316	46614	1380	158894	1444	138998	1508	373201	1572	54619	1636	59703	1700	66285	1764	53780	1828	81115	1892	68353	1956	158765	2020	49492
1061	1687711	1125	226092	1189	64119	1253	74570	1317	46039	1381	14533223	1445	196347	1509	491515	1573	90650	1637	59255	1701	161490	1765	66596	1829	116118	1893	242284	1957	1049164	2021	67209
1062	104184	1126	102337	1190	111923	1254	91763	1318	291342	1382	216658	1446	289808	1510	173525	1574	1126464	1638	212593	1702	97903	1766	846861	1830	58325	1894	114783	1958	111674	2022	358647
1063	134700	1127	211901	1191	227297	1255	51668	1319	107508	1383	259687	1447	197076	1511	532243	1575	212986	1639	240060	1703	28870	1767	369359	1831	56720	1895	72429	1959	55211	2023	154510
1064	499149	1128	81455	1192	64705	1256	293134	1320	114789	1384	114752	1448	504718	1512	156516	1576	221138	1640	63611	1704	98327	1768	18141	1832	81102	1896	151324	1960	511117	2024	93014
1065	99599	1129	386504	1193	176594	1257	324839	1321	57845	1385	962528	1449	50415	1513	119903	1577	81885	1641	94183	1705	114459	1769	163820	1833	154427	1897	74547	1961	49099	2025	125500
1066	324158	1130	258505	1194	141337	1258	265103	1322	110883	1386	2159586	1450	319742	1514	115811	1578	173517	1642	56848	1706	109322	1770	183742	1834	148114	1898	250119	1962	72991	2026	71112
1067	1388950	1131	112154	1195	37372	1259	72891	1323	125136	1387	774660	1451	65728	1515	157082	1579	339092	1643	117234	1707	183115	1771	34791	1835	104540	1899	277488	1963	98980	2027	137678
1068	68679	1132	30229	1196	67212	1260	162327	1324	75562	1388	232884	1452	79566	1516	45237	1580	18882	1644	56805	1708	132459	1772	140889	1836	74334	1900	72751	1964	44064	2028	99185
1069	168887	1133	57031	1197	43234	1261	313589	1325	105687	1389	62128	1453	3480717	1517	151063	1581	113134	1645	84443	1709	450136	1773	31744	1837	60710	1901	74547	1965	98573	2029	186923
1070	52836	1134	157451	1198	114725	1262	118350	1326	5185022	1390	86933	1454	744553	1518	109340	1582	704697	1646	245183	1710	61202	1774	316024	1838	250552	1902	45958	1966	55421	2030	112164
1071	532419	1135	136815	1199	49447	1263	119477	1327	70721	1391	101173	1455	215253	1519	478702	1583	140533	1647	202377	1711	61791	1775	56104	1839	33549	1903	38800	1967	94222	2031	173571
1072	176544	1136	54828	1200	5455040	1264	52966	1328	160396	1392	1920403	1456	767105	1520	70107	1584	50603	1648	667340	1712	100812	1776	189137	1840	179207	1904	132714	1968	75930	2032	843450
1073	30987	1137	607338	1201	102848	1265	127853	1329	142637	1393	145668	1457	166898	1521	186749	1585	85785	1649	212037	1713	127279	1777	25690	1841	1274843	1905	237707	1969	95095	2033	138090
1074	41563	1138	159209	1202	53729	1266	254999	1330	194158	1394	107269	1458	770263	1522	184177	1586	47666	1650	242488	1714	54171	1778	258022	1842	54397	1906	884035	1970	237168	2034	205091
1075	77017	1139	217738	1203	28034	1267	123134	1331	51317	1395	101985	1459	104240	1523	310139	1587	394121	1651	59927	1715	92086	1779	153047	1843	117204	1907	67754	1971	1877640	2035	389161
1076	74497	1140	102460	1204	184780	1268	127684	1332	50700	1396	81539	1460	134499	1524	79604	1588	64583	1652	95295	1716	364033	1780	259887	1844	111098	1908	65829	1972	235634	2036	128379
1077	81369	1141	124975	1205	63149	1269	143721	1333	84917	1397	68508	1461	103666	1525	141986	1589	54867	1653	142592	1717	53281	1781	60236	1845	42597	1909	159583	1973	650652	2037	201755
1078	824350	1142	340636	1206	235391	1270	288214	1334	81807	1398	57514	1462	83703	1526	81476	1590	843515	1654	195099	1718	70134	1782	143793	1846	175933	1910	84342	1974	71515	2038	86718
1079	90230	1143	85739	1207	209854	1271	180030	1335	66881	1399	115738	1463	253410	1527	153181	1591	224276	1655	152318	1719	247848	1783	61338	1847	100874	1911	459434	1975	73102	2039	348581
1080	73080	1144	135052	1208	54675	1272	46439	1336	39102	1400	203200	1464	90862	1528	192940	1592	90570	1656	422464	1720	173237	1784	564048	1848	491965	1912	1178794	1976	231099	2040	63099
1081	69049	1145	153227	1209	281449	1273	102969	1337	82328	1401	187063	1465	121616	1529	63019	1593	100842	1657	108109	1721	130117	1785	148889	1849	127348	1913	245542	1977	180049	2041	48240
1082	48455	1146	1744759	1210	171707	1274	182842	1338	39350	1402	97303	1466	160561	1530	243221	1594	18778	1658	75031	1722	223998	1786	154955	1850	264791	1914	36400	1978	55800	2042	94378
1083	79252	1147	82686	1211	35701	1275	508032	1339	77027	1403	125026	1467	174175	1531	150500	1595	182163	1659	112065	1723	4790305	1787	72262	1851	84010	1915	104072	1979	962481	2043	175809
1084	258487	1148	418012	1212	130248	1276	320662	1340	239970	1404	305847	1468	80342	1532	58971	1596	67925	1660	90778	1724	136671	1788	37644	1852	89883	1916	264786	1980	68525	2044	103320
1085	73674	1149	415337	1213	153734	1277	165717	1341	95529	1405	183467	1469	592012	1533	93015	1597	39253	1661	77071	1725	441416	1789	53226	1853	365415	1917	75960	1981	319735	2045	113937
1086	135129	1150	114434	1214	69362	1278	33170	1342	82327	1406	630172	1470	158632	1534	72452	1598	688110	1662	296334	1726	81867	1790	29975	1854	772531	1918	11141203	1982	48782	2046	114178
1087	170950	1151	133347	1215	170489	1279	73341	1343	211332	1407	141866	1471	101365	1535	65082	1599	213824	1663	77091	1727	261676	1791	178969	1855	227489	1919	163150	1983	218768	2047	77255
1088	427347	1152	135976	1216	74638	1280	588061	1344	136603	1408	94281	1472	46436	1536	1790298	1600	166321	1664	385822	1728	241831	1792	719459	1856	3331172	1920	47419	1984	78447	2048	145106

[a] ECFP4 2048-bit

Table S4. Similarity matrix of compound data sets computed as the median Tanimoto coefficient between its compounds using MACCS keys. [a]

Data set	BRD2	BRD3	BRD4	CREBBP	DNMT1	EHMT2	EP300	HDAC10	HDAC11	HDAC1	HDAC2	HDAC3	HDAC4	HDAC5	HDAC6	HDAC7	HDAC8	HDAC9	KAT2B	KDM1A	KDM4C	L3MBTL1	L3MBTL3	MAP3K7	MGEA5	NCOA1	NCOA3	PRMT1
**BRD2**	0.569	0.571	0.513	0.603	0.425	0.481	0.431	0.382	0.375	0.409	0.396	0.403	0.403	0.371	0.391	0.385	0.400	0.383	0.476	0.350	0.395	0.441	0.417	0.500	0.349	0.409	0.423	0.403
**BRD3**	0.571	0.574	0.514	0.603	0.427	0.481	0.435	0.385	0.377	0.412	0.397	0.405	0.405	0.374	0.393	0.386	0.404	0.385	0.478	0.352	0.397	0.443	0.418	0.500	0.350	0.410	0.424	0.405
**BRD4**	0.513	0.514	0.487	0.521	0.413	0.468	0.426	0.391	0.385	0.409	0.397	0.403	0.400	0.373	0.395	0.387	0.403	0.389	0.451	0.348	0.386	0.419	0.397	0.478	0.355	0.392	0.407	0.398
**CREBBP**	0.603	0.603	0.521	0.697	0.433	0.484	0.438	0.385	0.382	0.413	0.400	0.407	0.412	0.377	0.393	0.388	0.405	0.385	0.522	0.352	0.400	0.452	0.431	0.513	0.353	0.419	0.434	0.414
**DNMT1**	0.425	0.427	0.413	0.433	0.403	0.409	0.385	0.402	0.394	0.397	0.389	0.396	0.379	0.367	0.394	0.382	0.398	0.390	0.397	0.326	0.379	0.384	0.364	0.414	0.384	0.362	0.374	0.361
**EHMT2**	0.481	0.481	0.468	0.484	0.409	0.641	0.396	0.409	0.413	0.415	0.390	0.402	0.381	0.370	0.405	0.388	0.417	0.402	0.393	0.389	0.387	0.453	0.448	0.515	0.437	0.387	0.410	0.441
**EP300**	0.431	0.435	0.426	0.438	0.385	0.396	0.427	0.345	0.342	0.364	0.348	0.355	0.356	0.333	0.357	0.344	0.362	0.352	0.412	0.296	0.359	0.354	0.338	0.411	0.349	0.350	0.363	0.347
**HDAC10**	0.382	0.385	0.391	0.385	0.402	0.409	0.345	0.514	0.506	0.468	0.468	0.487	0.439	0.468	0.487	0.484	0.500	0.500	0.358	0.363	0.385	0.421	0.400	0.414	0.355	0.354	0.368	0.405
**HDAC11**	0.375	0.377	0.385	0.382	0.394	0.413	0.342	0.506	0.500	0.459	0.459	0.478	0.427	0.458	0.474	0.475	0.488	0.488	0.352	0.361	0.382	0.414	0.391	0.402	0.356	0.353	0.366	0.395
**HDAC1**	0.409	0.412	0.409	0.413	0.397	0.415	0.364	0.468	0.459	0.453	0.447	0.458	0.424	0.441	0.455	0.456	0.464	0.464	0.378	0.356	0.379	0.422	0.397	0.425	0.345	0.364	0.373	0.390
**HDAC2**	0.396	0.397	0.397	0.400	0.389	0.390	0.348	0.468	0.459	0.447	0.447	0.458	0.421	0.443	0.449	0.458	0.462	0.464	0.366	0.353	0.375	0.394	0.372	0.405	0.333	0.360	0.367	0.365
**HDAC3**	0.403	0.405	0.403	0.407	0.396	0.402	0.355	0.487	0.478	0.458	0.458	0.474	0.435	0.457	0.468	0.474	0.480	0.482	0.372	0.354	0.382	0.393	0.372	0.413	0.347	0.365	0.372	0.373
**HDAC4**	0.403	0.405	0.400	0.412	0.379	0.381	0.356	0.439	0.427	0.424	0.421	0.435	0.429	0.414	0.433	0.433	0.433	0.436	0.369	0.329	0.357	0.393	0.370	0.404	0.333	0.343	0.353	0.359
**HDAC5**	0.371	0.374	0.373	0.377	0.367	0.370	0.333	0.468	0.458	0.441	0.443	0.457	0.414	0.456	0.456	0.471	0.472	0.475	0.347	0.325	0.354	0.379	0.354	0.386	0.311	0.338	0.344	0.342
**HDAC6**	0.391	0.393	0.395	0.393	0.394	0.405	0.357	0.487	0.474	0.455	0.449	0.468	0.433	0.456	0.475	0.473	0.486	0.484	0.366	0.350	0.374	0.410	0.388	0.416	0.342	0.353	0.365	0.386
**HDAC7**	0.385	0.386	0.387	0.388	0.382	0.388	0.344	0.484	0.475	0.456	0.458	0.474	0.433	0.471	0.473	0.491	0.488	0.493	0.358	0.333	0.366	0.397	0.370	0.402	0.321	0.347	0.355	0.357
**HDAC8**	0.400	0.404	0.403	0.405	0.398	0.417	0.362	0.500	0.488	0.464	0.462	0.480	0.433	0.472	0.486	0.488	0.500	0.500	0.368	0.352	0.385	0.406	0.384	0.417	0.348	0.357	0.369	0.384
**HDAC9**	0.383	0.385	0.389	0.385	0.390	0.402	0.352	0.500	0.488	0.464	0.464	0.482	0.436	0.475	0.484	0.493	0.500	0.500	0.359	0.349	0.373	0.402	0.377	0.408	0.337	0.352	0.362	0.380
**KAT2B**	0.476	0.478	0.451	0.522	0.397	0.393	0.412	0.358	0.352	0.378	0.366	0.372	0.369	0.347	0.366	0.358	0.368	0.359	0.588	0.318	0.368	0.400	0.379	0.422	0.333	0.368	0.382	0.357
**KDM1A**	0.350	0.352	0.348	0.352	0.326	0.389	0.296	0.363	0.361	0.356	0.353	0.354	0.329	0.325	0.350	0.333	0.352	0.349	0.318	0.381	0.321	0.365	0.353	0.358	0.297	0.323	0.328	0.339
**KDM4C**	0.395	0.397	0.386	0.400	0.379	0.387	0.359	0.385	0.382	0.379	0.375	0.382	0.357	0.354	0.374	0.366	0.385	0.373	0.368	0.321	0.360	0.362	0.343	0.391	0.351	0.351	0.360	0.347
**L3MBTL1**	0.441	0.443	0.419	0.452	0.384	0.453	0.354	0.421	0.414	0.422	0.394	0.393	0.393	0.379	0.410	0.397	0.406	0.402	0.400	0.365	0.362	0.804	0.761	0.500	0.296	0.349	0.375	0.422
**L3MBTL3**	0.417	0.418	0.397	0.431	0.364	0.448	0.338	0.400	0.391	0.397	0.372	0.372	0.370	0.354	0.388	0.370	0.384	0.377	0.379	0.353	0.343	0.761	0.732	0.480	0.293	0.338	0.357	0.415
**MAP3K7**	0.500	0.500	0.478	0.513	0.414	0.515	0.411	0.414	0.402	0.425	0.405	0.413	0.404	0.386	0.416	0.402	0.417	0.408	0.422	0.358	0.391	0.500	0.480	0.541	0.381	0.397	0.409	0.421
**MGEA5**	0.349	0.350	0.355	0.353	0.384	0.437	0.349	0.355	0.356	0.345	0.333	0.347	0.333	0.311	0.342	0.321	0.348	0.337	0.333	0.297	0.351	0.296	0.293	0.381	0.687	0.333	0.352	0.350
**NCOA1**	0.409	0.410	0.392	0.419	0.362	0.387	0.350	0.354	0.353	0.364	0.360	0.365	0.343	0.338	0.353	0.347	0.357	0.352	0.368	0.323	0.351	0.349	0.338	0.397	0.333	0.352	0.359	0.345
**NCOA3**	0.423	0.424	0.407	0.434	0.374	0.410	0.363	0.368	0.366	0.373	0.367	0.372	0.353	0.344	0.365	0.355	0.369	0.362	0.382	0.328	0.360	0.375	0.357	0.409	0.352	0.359	0.369	0.357
**PRMT1**	0.403	0.405	0.398	0.414	0.361	0.441	0.347	0.405	0.395	0.390	0.365	0.373	0.359	0.342	0.386	0.357	0.384	0.380	0.357	0.339	0.347	0.422	0.415	0.421	0.350	0.345	0.357	0.397

[a] MACCS keys 166-bit

Table S5. Similarity matrix of compound data sets computed as Tanimoto coefficient between its DFP based on MACCS keys. [a]

Data set	BRD2	BRD3	BRD4	CREBBP	DNMT1	EHMT2	EP300	HDAC10	HDAC11	HDAC1	HDAC2	HDAC3	HDAC4	HDAC5	HDAC6	HDAC7	HDAC8	HDAC9	KAT2B	KDM1A	KDM4C	L3MBTL1	L3MBTL3	MAP3K7	MGEA5	NCOA1	NCOA3	PRMT1
**BRD2**	1.000	1.000	0.852	0.981	0.585	0.533	0.559	0.392	0.384	0.444	0.375	0.397	0.439	0.333	0.365	0.315	0.392	0.356	0.678	0.313	0.567	0.475	0.475	0.600	0.329	0.526	0.586	0.403
**BRD3**	1.000	1.000	0.852	0.981	0.585	0.533	0.559	0.392	0.384	0.444	0.375	0.397	0.439	0.333	0.365	0.315	0.392	0.356	0.678	0.313	0.567	0.475	0.475	0.600	0.329	0.526	0.586	0.403
**BRD4**	0.852	0.852	1.000	0.833	0.617	0.603	0.587	0.406	0.397	0.485	0.409	0.433	0.436	0.387	0.397	0.364	0.406	0.388	0.661	0.368	0.630	0.500	0.500	0.631	0.338	0.620	0.686	0.467
**CREBBP**	0.981	0.981	0.833	1.000	0.569	0.520	0.567	0.378	0.370	0.431	0.361	0.384	0.424	0.319	0.351	0.301	0.378	0.343	0.690	0.297	0.576	0.459	0.459	0.586	0.333	0.536	0.597	0.388
**DNMT1**	0.585	0.585	0.617	0.569	1.000	0.514	0.561	0.471	0.485	0.508	0.433	0.500	0.438	0.413	0.463	0.388	0.471	0.433	0.524	0.421	0.625	0.475	0.475	0.603	0.508	0.556	0.679	0.444
**EHMT2**	0.533	0.533	0.603	0.520	0.514	1.000	0.474	0.418	0.392	0.468	0.385	0.423	0.351	0.329	0.410	0.364	0.418	0.385	0.403	0.388	0.411	0.478	0.478	0.658	0.392	0.412	0.530	0.585
**EP300**	0.559	0.559	0.587	0.567	0.561	0.474	1.000	0.392	0.384	0.465	0.394	0.397	0.418	0.353	0.384	0.333	0.392	0.375	0.623	0.313	0.567	0.385	0.385	0.514	0.464	0.554	0.643	0.403
**HDAC10**	0.392	0.392	0.406	0.378	0.471	0.418	0.392	1.000	0.815	0.870	0.882	0.904	0.643	0.745	0.885	0.860	0.852	0.846	0.333	0.446	0.444	0.475	0.475	0.473	0.307	0.424	0.391	0.492
**HDAC11**	0.384	0.384	0.397	0.370	0.485	0.392	0.384	0.815	1.000	0.768	0.843	0.796	0.579	0.776	0.811	0.784	0.849	0.774	0.324	0.463	0.483	0.393	0.393	0.446	0.315	0.414	0.403	0.459
**HDAC1**	0.444	0.444	0.485	0.431	0.508	0.468	0.465	0.870	0.768	1.000	0.865	0.852	0.632	0.765	0.868	0.808	0.836	0.796	0.406	0.491	0.533	0.492	0.492	0.528	0.320	0.491	0.475	0.559
**HDAC2**	0.375	0.375	0.409	0.361	0.433	0.385	0.394	0.882	0.843	0.865	1.000	0.900	0.571	0.848	0.808	0.854	0.846	0.878	0.333	0.481	0.500	0.431	0.431	0.438	0.270	0.482	0.417	0.475
**HDAC3**	0.397	0.397	0.433	0.384	0.500	0.423	0.397	0.904	0.796	0.852	0.900	1.000	0.569	0.796	0.865	0.840	0.800	0.827	0.377	0.455	0.500	0.458	0.458	0.460	0.311	0.456	0.419	0.475
**HDAC4**	0.439	0.439	0.436	0.424	0.438	0.351	0.418	0.643	0.579	0.632	0.571	0.569	1.000	0.588	0.636	0.635	0.586	0.571	0.419	0.431	0.431	0.436	0.436	0.464	0.304	0.434	0.397	0.482
**HDAC5**	0.333	0.333	0.387	0.319	0.413	0.329	0.353	0.745	0.776	0.765	0.848	0.796	0.588	1.000	0.776	0.864	0.780	0.809	0.328	0.458	0.482	0.434	0.434	0.441	0.261	0.490	0.418	0.429
**HDAC6**	0.365	0.365	0.397	0.351	0.463	0.410	0.384	0.885	0.811	0.868	0.808	0.865	0.636	0.776	1.000	0.896	0.849	0.843	0.343	0.463	0.459	0.491	0.491	0.466	0.315	0.414	0.403	0.535
**HDAC7**	0.315	0.315	0.364	0.301	0.388	0.364	0.333	0.860	0.784	0.808	0.854	0.840	0.635	0.864	0.896	1.000	0.824	0.894	0.309	0.480	0.424	0.455	0.455	0.437	0.282	0.426	0.390	0.500
**HDAC8**	0.392	0.392	0.406	0.378	0.471	0.418	0.392	0.852	0.849	0.836	0.846	0.800	0.586	0.780	0.849	0.824	1.000	0.846	0.333	0.446	0.468	0.426	0.426	0.473	0.307	0.448	0.413	0.517
**HDAC9**	0.356	0.356	0.388	0.343	0.433	0.385	0.375	0.846	0.774	0.796	0.878	0.827	0.571	0.809	0.843	0.894	0.846	1.000	0.333	0.453	0.450	0.431	0.431	0.458	0.288	0.482	0.417	0.450
**KAT2B**	0.678	0.678	0.661	0.690	0.524	0.403	0.623	0.333	0.324	0.406	0.333	0.377	0.419	0.328	0.343	0.309	0.333	0.333	1.000	0.305	0.526	0.383	0.383	0.500	0.382	0.509	0.574	0.339
**KDM1A**	0.313	0.313	0.368	0.297	0.421	0.388	0.313	0.446	0.463	0.491	0.481	0.455	0.431	0.458	0.463	0.480	0.446	0.453	0.305	1.000	0.385	0.447	0.447	0.406	0.254	0.477	0.458	0.500
**KDM4C**	0.567	0.567	0.630	0.576	0.625	0.411	0.567	0.444	0.483	0.533	0.500	0.500	0.431	0.482	0.459	0.424	0.468	0.450	0.526	0.385	1.000	0.418	0.418	0.563	0.413	0.667	0.667	0.390
**L3MBTL1**	0.475	0.475	0.500	0.459	0.475	0.478	0.385	0.475	0.393	0.492	0.431	0.458	0.436	0.434	0.491	0.455	0.426	0.431	0.383	0.447	0.418	1.000	1.000	0.600	0.288	0.420	0.462	0.500
**L3MBTL3**	0.475	0.475	0.500	0.459	0.475	0.478	0.385	0.475	0.393	0.492	0.431	0.458	0.436	0.434	0.491	0.455	0.426	0.431	0.383	0.447	0.418	1.000	1.000	0.600	0.288	0.420	0.462	0.500
**MAP3K7**	0.600	0.600	0.631	0.586	0.603	0.658	0.514	0.473	0.446	0.528	0.438	0.460	0.464	0.441	0.466	0.437	0.473	0.458	0.500	0.406	0.563	0.600	0.600	1.000	0.408	0.576	0.607	0.515
**MGEA5**	0.329	0.329	0.338	0.333	0.508	0.392	0.464	0.307	0.315	0.320	0.270	0.311	0.304	0.261	0.315	0.282	0.307	0.288	0.382	0.254	0.413	0.288	0.288	0.408	1.000	0.323	0.426	0.349
**NCOA1**	0.526	0.526	0.620	0.536	0.556	0.412	0.554	0.424	0.414	0.491	0.482	0.456	0.434	0.490	0.414	0.426	0.448	0.482	0.509	0.477	0.667	0.420	0.420	0.576	0.323	1.000	0.781	0.389
**NCOA3**	0.586	0.586	0.686	0.597	0.679	0.530	0.643	0.391	0.403	0.475	0.417	0.419	0.397	0.418	0.403	0.390	0.413	0.417	0.574	0.458	0.667	0.462	0.462	0.607	0.426	0.781	1.000	0.455
**PRMT1**	0.403	0.403	0.467	0.388	0.444	0.585	0.403	0.492	0.459	0.559	0.475	0.475	0.482	0.429	0.535	0.500	0.517	0.450	0.339	0.500	0.390	0.500	0.500	0.515	0.349	0.389	0.455	1.000

[a] MACCS keys 166-bit

Table S6. Similarity matrix of compound data sets computed as Tanimoto coefficient between its SB-DFP based on MACCS keys. [a]

Data set	BRD2	BRD3	BRD4	CREBBP	DNMT1	EHMT2	EP300	HDAC10	HDAC11	HDAC1	HDAC2	HDAC3	HDAC4	HDAC5	HDAC6	HDAC7	HDAC8	HDAC9	KAT2B	KDM1A	KDM4C	L3MBTL1	L3MBTL3	MAP3K7	MGEA5	NCOA1	NCOA3	PRMT1
**BRD2**	1.000	0.918	0.816	0.746	0.277	0.323	0.281	0.215	0.198	0.327	0.263	0.301	0.349	0.224	0.240	0.218	0.294	0.247	0.405	0.153	0.193	0.300	0.275	0.302	0.225	0.181	0.208	0.189
**BRD3**	0.918	1.000	0.895	0.685	0.337	0.316	0.344	0.227	0.211	0.333	0.273	0.309	0.356	0.238	0.262	0.232	0.302	0.259	0.427	0.182	0.207	0.310	0.286	0.311	0.211	0.197	0.222	0.202
**BRD4**	0.816	0.895	1.000	0.592	0.343	0.309	0.366	0.206	0.190	0.340	0.253	0.302	0.348	0.213	0.243	0.207	0.281	0.235	0.369	0.186	0.225	0.256	0.262	0.303	0.228	0.203	0.228	0.194
**CREBBP**	0.746	0.685	0.592	1.000	0.217	0.293	0.191	0.143	0.150	0.242	0.184	0.238	0.253	0.169	0.167	0.182	0.214	0.179	0.424	0.095	0.143	0.299	0.269	0.338	0.211	0.133	0.169	0.154
**DNMT1**	0.277	0.337	0.343	0.217	1.000	0.168	0.457	0.350	0.300	0.421	0.369	0.349	0.337	0.333	0.425	0.364	0.390	0.358	0.293	0.148	0.370	0.138	0.128	0.244	0.333	0.212	0.313	0.244
**EHMT2**	0.323	0.316	0.309	0.293	0.168	1.000	0.217	0.159	0.167	0.202	0.160	0.196	0.129	0.079	0.217	0.050	0.214	0.090	0.205	0.160	0.119	0.348	0.338	0.295	0.256	0.088	0.139	0.200
**EP300**	0.281	0.344	0.366	0.191	0.457	0.217	1.000	0.186	0.181	0.253	0.253	0.279	0.265	0.224	0.297	0.235	0.286	0.250	0.351	0.192	0.253	0.094	0.096	0.217	0.273	0.213	0.323	0.215
**HDAC10**	0.215	0.227	0.206	0.143	0.350	0.159	0.186	1.000	0.726	0.535	0.547	0.539	0.439	0.440	0.636	0.542	0.750	0.531	0.169	0.283	0.333	0.221	0.141	0.182	0.158	0.231	0.263	0.265
**HDAC11**	0.198	0.211	0.190	0.150	0.300	0.167	0.181	0.726	1.000	0.458	0.462	0.477	0.400	0.478	0.576	0.522	0.709	0.544	0.178	0.237	0.312	0.234	0.149	0.176	0.183	0.200	0.214	0.206
**HDAC1**	0.327	0.333	0.340	0.242	0.421	0.202	0.253	0.535	0.458	1.000	0.681	0.603	0.514	0.391	0.645	0.422	0.597	0.460	0.244	0.288	0.347	0.191	0.153	0.333	0.193	0.246	0.254	0.226
**HDAC2**	0.263	0.273	0.253	0.184	0.369	0.160	0.253	0.547	0.462	0.681	1.000	0.698	0.500	0.411	0.643	0.473	0.591	0.491	0.213	0.292	0.300	0.184	0.128	0.256	0.173	0.246	0.295	0.274
**HDAC3**	0.301	0.309	0.302	0.238	0.349	0.196	0.279	0.539	0.477	0.603	0.698	1.000	0.493	0.429	0.568	0.439	0.631	0.509	0.256	0.269	0.314	0.230	0.171	0.303	0.247	0.263	0.290	0.270
**HDAC4**	0.349	0.356	0.348	0.253	0.337	0.129	0.265	0.439	0.400	0.514	0.500	0.493	1.000	0.442	0.423	0.453	0.485	0.500	0.240	0.194	0.339	0.194	0.120	0.270	0.264	0.218	0.210	0.271
**HDAC5**	0.224	0.238	0.213	0.169	0.333	0.079	0.224	0.440	0.478	0.391	0.411	0.429	0.442	1.000	0.397	0.800	0.472	0.833	0.228	0.163	0.333	0.167	0.070	0.203	0.193	0.419	0.333	0.179
**HDAC6**	0.240	0.262	0.243	0.167	0.425	0.217	0.297	0.636	0.576	0.645	0.643	0.568	0.423	0.397	1.000	0.429	0.754	0.468	0.178	0.292	0.333	0.222	0.169	0.216	0.182	0.250	0.275	0.244
**HDAC7**	0.218	0.232	0.207	0.182	0.364	0.050	0.235	0.542	0.522	0.422	0.473	0.439	0.453	0.800	0.429	1.000	0.509	0.839	0.200	0.180	0.375	0.161	0.068	0.197	0.167	0.353	0.317	0.193
**HDAC8**	0.294	0.302	0.281	0.214	0.390	0.214	0.286	0.750	0.709	0.597	0.591	0.631	0.485	0.472	0.754	0.509	1.000	0.558	0.215	0.318	0.364	0.236	0.160	0.244	0.221	0.296	0.322	0.278
**HDAC9**	0.247	0.259	0.235	0.179	0.358	0.090	0.250	0.531	0.544	0.460	0.491	0.509	0.500	0.833	0.468	0.839	0.558	1.000	0.217	0.200	0.367	0.179	0.085	0.213	0.203	0.424	0.342	0.211
**KAT2B**	0.405	0.427	0.369	0.424	0.293	0.205	0.351	0.169	0.178	0.244	0.213	0.256	0.240	0.228	0.178	0.200	0.215	0.217	1.000	0.119	0.155	0.246	0.179	0.271	0.229	0.192	0.228	0.183
**KDM1A**	0.153	0.182	0.186	0.095	0.148	0.160	0.192	0.283	0.237	0.288	0.292	0.269	0.194	0.163	0.292	0.180	0.318	0.200	0.119	1.000	0.150	0.153	0.158	0.226	0.106	0.290	0.239	0.291
**KDM4C**	0.193	0.207	0.225	0.143	0.370	0.119	0.253	0.333	0.312	0.347	0.300	0.314	0.339	0.333	0.333	0.375	0.364	0.367	0.155	0.150	1.000	0.103	0.090	0.186	0.312	0.333	0.306	0.182
**L3MBTL1**	0.300	0.310	0.256	0.299	0.138	0.348	0.094	0.221	0.234	0.191	0.184	0.230	0.194	0.167	0.222	0.161	0.236	0.179	0.246	0.153	0.103	1.000	0.800	0.323	0.162	0.100	0.086	0.262
**L3MBTL3**	0.275	0.286	0.262	0.269	0.128	0.338	0.096	0.141	0.149	0.153	0.128	0.171	0.120	0.070	0.169	0.068	0.160	0.085	0.179	0.158	0.090	0.800	1.000	0.333	0.149	0.104	0.089	0.293
**MAP3K7**	0.302	0.311	0.303	0.338	0.244	0.295	0.217	0.182	0.176	0.333	0.256	0.303	0.270	0.203	0.216	0.197	0.244	0.213	0.271	0.226	0.186	0.323	0.333	1.000	0.261	0.235	0.224	0.250
**MGEA5**	0.225	0.211	0.228	0.211	0.333	0.256	0.273	0.158	0.183	0.193	0.173	0.247	0.264	0.193	0.182	0.167	0.221	0.203	0.229	0.106	0.312	0.162	0.149	0.261	1.000	0.132	0.214	0.188
**NCOA1**	0.181	0.197	0.203	0.133	0.212	0.088	0.213	0.231	0.200	0.246	0.246	0.263	0.218	0.419	0.250	0.353	0.296	0.424	0.192	0.290	0.333	0.100	0.104	0.235	0.132	1.000	0.517	0.184
**NCOA3**	0.208	0.222	0.228	0.169	0.313	0.139	0.323	0.263	0.214	0.254	0.295	0.290	0.210	0.333	0.275	0.317	0.322	0.342	0.228	0.239	0.306	0.086	0.089	0.224	0.214	0.517	1.000	0.222
**PRMT1**	0.189	0.202	0.194	0.154	0.244	0.200	0.215	0.265	0.206	0.226	0.274	0.270	0.271	0.179	0.244	0.193	0.278	0.211	0.183	0.291	0.182	0.262	0.293	0.250	0.188	0.184	0.222	1.000

[a] MACCS keys 166-bit

Table S7. Similarity matrix of compound data sets computed as the median Tanimoto coefficient between its compounds using ECFP4. [a]

Data set	BRD2	BRD3	BRD4	CREBBP	DNMT1	EHMT2	EP300	HDAC10	HDAC11	HDAC1	HDAC2	HDAC3	HDAC4	HDAC5	HDAC6	HDAC7	HDAC8	HDAC9	KAT2B	KDM1A	KDM4C	L3MBTL1	L3MBTL3	MAP3K7	MGEA5	NCOA1	NCOA3	PRMT1
**BRD2**	0.152	0.152	0.136	0.165	0.100	0.109	0.099	0.096	0.098	0.101	0.099	0.098	0.101	0.101	0.098	0.100	0.103	0.100	0.114	0.103	0.096	0.102	0.099	0.117	0.067	0.102	0.103	0.085
**BRD3**	0.152	0.153	0.137	0.165	0.100	0.110	0.099	0.096	0.099	0.101	0.099	0.098	0.101	0.101	0.098	0.100	0.103	0.100	0.114	0.104	0.096	0.103	0.100	0.117	0.067	0.102	0.103	0.085
**BRD4**	0.136	0.137	0.133	0.132	0.101	0.107	0.099	0.100	0.101	0.105	0.103	0.102	0.105	0.103	0.102	0.102	0.105	0.103	0.109	0.102	0.097	0.103	0.100	0.117	0.068	0.103	0.104	0.087
**CREBBP**	0.165	0.165	0.132	0.287	0.095	0.108	0.099	0.092	0.094	0.095	0.093	0.093	0.094	0.096	0.092	0.096	0.097	0.094	0.124	0.098	0.095	0.090	0.091	0.112	0.065	0.099	0.099	0.081
**DNMT1**	0.100	0.100	0.101	0.095	0.116	0.089	0.102	0.116	0.117	0.120	0.119	0.115	0.113	0.119	0.117	0.120	0.118	0.121	0.102	0.106	0.105	0.103	0.098	0.108	0.072	0.107	0.109	0.087
**EHMT2**	0.109	0.110	0.107	0.108	0.089	0.232	0.088	0.091	0.096	0.093	0.087	0.090	0.089	0.092	0.093	0.087	0.095	0.092	0.091	0.100	0.087	0.115	0.118	0.111	0.075	0.092	0.097	0.088
**EP300**	0.099	0.099	0.099	0.099	0.102	0.088	0.108	0.099	0.101	0.104	0.102	0.100	0.100	0.103	0.101	0.102	0.102	0.102	0.108	0.097	0.099	0.087	0.088	0.103	0.068	0.100	0.102	0.083
**HDAC10**	0.096	0.096	0.100	0.092	0.116	0.091	0.099	0.167	0.159	0.148	0.147	0.148	0.133	0.153	0.152	0.161	0.155	0.162	0.098	0.112	0.111	0.111	0.105	0.105	0.070	0.107	0.108	0.096
**HDAC11**	0.098	0.099	0.101	0.094	0.117	0.096	0.101	0.159	0.157	0.144	0.144	0.143	0.132	0.150	0.147	0.157	0.150	0.158	0.099	0.115	0.113	0.115	0.111	0.105	0.068	0.109	0.110	0.095
**HDAC1**	0.101	0.101	0.105	0.095	0.120	0.093	0.104	0.148	0.144	0.149	0.148	0.145	0.134	0.147	0.147	0.151	0.147	0.154	0.103	0.115	0.112	0.113	0.108	0.111	0.069	0.113	0.114	0.098
**HDAC2**	0.099	0.099	0.103	0.093	0.119	0.087	0.102	0.147	0.144	0.148	0.149	0.146	0.136	0.147	0.147	0.152	0.148	0.153	0.101	0.115	0.110	0.115	0.108	0.110	0.068	0.112	0.113	0.094
**HDAC3**	0.098	0.098	0.102	0.093	0.115	0.090	0.100	0.148	0.143	0.145	0.146	0.147	0.134	0.145	0.146	0.151	0.147	0.153	0.102	0.111	0.108	0.111	0.105	0.109	0.070	0.110	0.111	0.093
**HDAC4**	0.101	0.101	0.105	0.094	0.113	0.089	0.100	0.133	0.132	0.134	0.136	0.134	0.135	0.131	0.135	0.135	0.133	0.138	0.095	0.110	0.106	0.124	0.116	0.110	0.078	0.110	0.110	0.094
**HDAC5**	0.101	0.101	0.103	0.096	0.119	0.092	0.103	0.153	0.150	0.147	0.147	0.145	0.131	0.154	0.148	0.158	0.152	0.159	0.105	0.117	0.113	0.114	0.109	0.107	0.067	0.114	0.114	0.095
**HDAC6**	0.098	0.098	0.102	0.092	0.117	0.093	0.101	0.152	0.147	0.147	0.147	0.146	0.135	0.148	0.149	0.153	0.150	0.156	0.101	0.114	0.111	0.114	0.108	0.106	0.071	0.111	0.112	0.096
**HDAC7**	0.100	0.100	0.102	0.096	0.120	0.087	0.102	0.161	0.157	0.151	0.152	0.151	0.135	0.158	0.153	0.165	0.158	0.167	0.101	0.116	0.113	0.111	0.107	0.104	0.068	0.113	0.113	0.096
**HDAC8**	0.103	0.103	0.105	0.097	0.118	0.095	0.102	0.155	0.150	0.147	0.148	0.147	0.133	0.152	0.150	0.158	0.153	0.159	0.100	0.116	0.110	0.115	0.110	0.108	0.067	0.110	0.111	0.095
**HDAC9**	0.100	0.100	0.103	0.094	0.121	0.092	0.102	0.162	0.158	0.154	0.153	0.153	0.138	0.159	0.156	0.167	0.159	0.169	0.101	0.115	0.114	0.114	0.108	0.106	0.067	0.114	0.114	0.098
**KAT2B**	0.114	0.114	0.109	0.124	0.102	0.091	0.108	0.098	0.099	0.103	0.101	0.102	0.095	0.105	0.101	0.101	0.100	0.101	0.181	0.091	0.101	0.091	0.091	0.100	0.062	0.103	0.105	0.082
**KDM1A**	0.103	0.104	0.102	0.098	0.106	0.100	0.097	0.112	0.115	0.115	0.115	0.111	0.110	0.117	0.114	0.116	0.116	0.115	0.091	0.143	0.101	0.111	0.107	0.103	0.075	0.106	0.105	0.088
**KDM4C**	0.096	0.096	0.097	0.095	0.105	0.087	0.099	0.111	0.113	0.112	0.110	0.108	0.106	0.113	0.111	0.113	0.110	0.114	0.101	0.101	0.101	0.101	0.094	0.100	0.065	0.101	0.103	0.083
**L3MBTL1**	0.102	0.103	0.103	0.090	0.103	0.115	0.087	0.111	0.115	0.113	0.115	0.111	0.124	0.114	0.114	0.111	0.115	0.114	0.091	0.111	0.101	0.553	0.490	0.143	0.061	0.097	0.102	0.079
**L3MBTL3**	0.099	0.100	0.100	0.091	0.098	0.118	0.088	0.105	0.111	0.108	0.108	0.105	0.116	0.109	0.108	0.107	0.110	0.108	0.091	0.107	0.094	0.490	0.409	0.123	0.059	0.093	0.097	0.077
**MAP3K7**	0.117	0.117	0.117	0.112	0.108	0.111	0.103	0.105	0.105	0.111	0.110	0.109	0.110	0.107	0.106	0.104	0.108	0.106	0.100	0.103	0.100	0.143	0.123	0.138	0.083	0.104	0.108	0.089
**MGEA5**	0.067	0.067	0.068	0.065	0.072	0.075	0.068	0.070	0.068	0.069	0.068	0.070	0.078	0.067	0.071	0.068	0.067	0.067	0.062	0.075	0.065	0.061	0.059	0.083	0.321	0.068	0.069	0.067
**NCOA1**	0.102	0.102	0.103	0.099	0.107	0.092	0.100	0.107	0.109	0.113	0.112	0.110	0.110	0.114	0.111	0.113	0.110	0.114	0.103	0.106	0.101	0.097	0.093	0.104	0.068	0.107	0.108	0.084
**NCOA3**	0.103	0.103	0.104	0.099	0.109	0.097	0.102	0.108	0.110	0.114	0.113	0.111	0.110	0.114	0.112	0.113	0.111	0.114	0.105	0.105	0.103	0.102	0.097	0.108	0.069	0.108	0.109	0.086
**PRMT1**	0.085	0.085	0.087	0.081	0.087	0.088	0.083	0.096	0.095	0.098	0.094	0.093	0.094	0.095	0.096	0.096	0.095	0.098	0.082	0.088	0.083	0.079	0.077	0.089	0.067	0.084	0.086	0.077

[a] ECFP4 2048-bit

Table S8. Similarity matrix of compound data sets computed as Tanimoto coefficient between its DFP based on ECFP4. [a]

Data set	BRD2	BRD3	BRD4	CREBBP	DNMT1	EHMT2	EP300	HDAC10	HDAC11	HDAC1	HDAC2	HDAC3	HDAC4	HDAC5	HDAC6	HDAC7	HDAC8	HDAC9	KAT2B	KDM1A	KDM4C	L3MBTL1	L3MBTL3	MAP3K7	MGEA5	NCOA1	NCOA3	PRMT1
**BRD2**	1.000	0.963	0.367	0.703	0.177	0.153	0.267	0.100	0.162	0.200	0.143	0.177	0.194	0.143	0.143	0.114	0.147	0.143	0.250	0.143	0.194	0.125	0.128	0.192	0.095	0.226	0.194	0.200
**BRD3**	0.963	1.000	0.379	0.722	0.182	0.155	0.276	0.103	0.167	0.206	0.147	0.182	0.200	0.147	0.147	0.118	0.152	0.147	0.258	0.146	0.200	0.128	0.130	0.196	0.098	0.233	0.200	0.207
**BRD4**	0.367	0.379	1.000	0.282	0.350	0.196	0.471	0.192	0.304	0.381	0.286	0.350	0.412	0.286	0.286	0.238	0.300	0.286	0.421	0.250	0.412	0.206	0.212	0.290	0.138	0.471	0.333	0.438
**CREBBP**	0.703	0.722	0.282	1.000	0.140	0.149	0.205	0.082	0.130	0.159	0.114	0.140	0.150	0.114	0.114	0.091	0.116	0.114	0.225	0.140	0.150	0.105	0.107	0.164	0.078	0.175	0.150	0.154
**DNMT1**	0.177	0.182	0.350	0.140	1.000	0.125	0.500	0.429	0.526	0.647	0.529	0.625	0.643	0.625	0.625	0.563	0.667	0.625	0.300	0.478	0.643	0.177	0.182	0.263	0.185	0.714	0.643	0.467
**EHMT2**	0.153	0.155	0.196	0.149	0.125	1.000	0.156	0.115	0.140	0.143	0.102	0.125	0.133	0.102	0.125	0.082	0.104	0.125	0.102	0.127	0.133	0.133	0.136	0.188	0.091	0.130	0.109	0.163
**EP300**	0.267	0.276	0.471	0.205	0.500	0.156	1.000	0.273	0.421	0.625	0.500	0.500	0.500	0.500	0.412	0.438	0.438	0.412	0.412	0.333	0.500	0.152	0.156	0.243	0.154	0.571	0.615	0.539
**HDAC10**	0.100	0.103	0.192	0.082	0.429	0.115	0.273	1.000	0.650	0.524	0.579	0.579	0.421	0.579	0.667	0.611	0.611	0.667	0.154	0.310	0.421	0.158	0.162	0.156	0.091	0.400	0.421	0.300
**HDAC11**	0.162	0.167	0.304	0.130	0.526	0.140	0.421	0.650	1.000	0.722	0.706	0.611	0.529	0.706	0.813	0.750	0.647	0.706	0.261	0.423	0.529	0.162	0.167	0.186	0.129	0.500	0.529	0.389
**HDAC1**	0.200	0.206	0.381	0.159	0.647	0.143	0.625	0.524	0.722	1.000	0.867	0.867	0.667	0.867	0.750	0.800	0.800	0.750	0.273	0.440	0.667	0.167	0.171	0.250	0.172	0.733	0.667	0.500
**HDAC2**	0.143	0.147	0.286	0.114	0.529	0.102	0.500	0.579	0.706	0.867	1.000	0.857	0.643	0.857	0.733	0.923	0.786	0.733	0.182	0.417	0.643	0.177	0.182	0.200	0.143	0.600	0.643	0.467
**HDAC3**	0.177	0.182	0.350	0.140	0.625	0.125	0.500	0.579	0.611	0.867	0.857	1.000	0.643	0.857	0.733	0.786	0.923	0.857	0.238	0.360	0.643	0.177	0.182	0.231	0.143	0.714	0.643	0.467
**HDAC4**	0.194	0.200	0.412	0.150	0.643	0.133	0.500	0.421	0.529	0.667	0.643	0.643	1.000	0.533	0.643	0.571	0.571	0.533	0.278	0.409	1.000	0.194	0.200	0.216	0.208	0.750	0.818	0.727
**HDAC5**	0.143	0.147	0.286	0.114	0.625	0.102	0.500	0.579	0.706	0.867	0.857	0.857	0.533	1.000	0.733	0.923	0.923	0.857	0.238	0.417	0.533	0.177	0.182	0.200	0.103	0.600	0.533	0.375
**HDAC6**	0.143	0.147	0.286	0.114	0.625	0.125	0.412	0.667	0.813	0.750	0.733	0.733	0.643	0.733	1.000	0.786	0.786	0.857	0.238	0.417	0.643	0.177	0.182	0.200	0.143	0.600	0.643	0.467
**HDAC7**	0.114	0.118	0.238	0.091	0.563	0.082	0.438	0.611	0.750	0.800	0.923	0.786	0.571	0.923	0.786	1.000	0.846	0.786	0.191	0.435	0.571	0.182	0.188	0.175	0.107	0.533	0.571	0.400
**HDAC8**	0.147	0.152	0.300	0.116	0.667	0.104	0.438	0.611	0.647	0.800	0.786	0.923	0.571	0.923	0.786	0.846	1.000	0.923	0.250	0.375	0.571	0.182	0.188	0.205	0.107	0.643	0.571	0.400
**HDAC9**	0.143	0.147	0.286	0.114	0.625	0.125	0.412	0.667	0.706	0.750	0.733	0.857	0.533	0.857	0.857	0.786	0.923	1.000	0.238	0.360	0.533	0.177	0.182	0.200	0.103	0.600	0.533	0.375
**KAT2B**	0.250	0.258	0.421	0.225	0.300	0.102	0.412	0.154	0.261	0.273	0.182	0.238	0.278	0.238	0.238	0.191	0.250	0.238	1.000	0.172	0.278	0.143	0.147	0.171	0.103	0.333	0.278	0.294
**KDM1A**	0.143	0.146	0.250	0.140	0.478	0.127	0.333	0.310	0.423	0.440	0.417	0.360	0.409	0.417	0.417	0.435	0.375	0.360	0.172	1.000	0.409	0.200	0.205	0.217	0.143	0.391	0.409	0.304
**KDM4C**	0.194	0.200	0.412	0.150	0.643	0.133	0.500	0.421	0.529	0.667	0.643	0.643	1.000	0.533	0.643	0.571	0.571	0.533	0.278	0.409	1.000	0.194	0.200	0.216	0.208	0.750	0.818	0.727
**L3MBTL1**	0.125	0.128	0.206	0.105	0.177	0.133	0.152	0.158	0.162	0.167	0.177	0.177	0.194	0.177	0.177	0.182	0.182	0.177	0.143	0.200	0.194	1.000	0.963	0.240	0.070	0.188	0.194	0.161
**L3MBTL3**	0.128	0.130	0.212	0.107	0.182	0.136	0.156	0.162	0.167	0.171	0.182	0.182	0.200	0.182	0.182	0.188	0.188	0.182	0.147	0.205	0.200	0.963	1.000	0.245	0.071	0.194	0.200	0.167
**MAP3K7**	0.192	0.196	0.290	0.164	0.263	0.188	0.243	0.156	0.186	0.250	0.200	0.231	0.216	0.200	0.200	0.175	0.205	0.200	0.171	0.217	0.216	0.240	0.245	1.000	0.125	0.278	0.250	0.189
**MGEA5**	0.095	0.098	0.138	0.078	0.185	0.091	0.154	0.091	0.129	0.172	0.143	0.143	0.208	0.103	0.143	0.107	0.107	0.103	0.103	0.143	0.208	0.070	0.071	0.125	1.000	0.154	0.208	0.217
**NCOA1**	0.226	0.233	0.471	0.175	0.714	0.130	0.571	0.400	0.500	0.733	0.600	0.714	0.750	0.600	0.600	0.533	0.643	0.600	0.333	0.391	0.750	0.188	0.194	0.278	0.154	1.000	0.750	0.539
**NCOA3**	0.194	0.200	0.333	0.150	0.643	0.109	0.615	0.421	0.529	0.667	0.643	0.643	0.818	0.533	0.643	0.571	0.571	0.533	0.278	0.409	0.818	0.194	0.200	0.250	0.208	0.750	1.000	0.583
**PRMT1**	0.200	0.207	0.438	0.154	0.467	0.163	0.539	0.300	0.389	0.500	0.467	0.467	0.727	0.375	0.467	0.400	0.400	0.375	0.294	0.304	0.727	0.161	0.167	0.189	0.217	0.539	0.583	1.000

[a] ECFP4 2048-bit

Table S9. Similarity matrix of compound data sets computed as Tanimoto coefficient between its SB-DFP based on ECFP4. [a]

Data set	BRD2	BRD3	BRD4	CREBBP	DNMT1	EHMT2	EP300	HDAC10	HDAC11	HDAC1	HDAC2	HDAC3	HDAC4	HDAC5	HDAC6	HDAC7	HDAC8	HDAC9	KAT2B	KDM1A	KDM4C	L3MBTL1	L3MBTL3	MAP3K7	MGEA5	NCOA1	NCOA3	PRMT1
**BRD2**	1.000	0.933	0.519	0.489	0.097	0.070	0.073	0.082	0.078	0.103	0.101	0.089	0.082	0.074	0.079	0.079	0.102	0.069	0.072	0.078	0.052	0.036	0.040	0.106	0.041	0.026	0.051	0.088
**BRD3**	0.933	1.000	0.537	0.475	0.101	0.070	0.075	0.089	0.082	0.106	0.105	0.093	0.084	0.080	0.082	0.086	0.107	0.074	0.075	0.077	0.049	0.040	0.040	0.102	0.044	0.028	0.048	0.088
**BRD4**	0.519	0.537	1.000	0.378	0.116	0.087	0.098	0.098	0.085	0.146	0.130	0.124	0.107	0.085	0.118	0.093	0.132	0.090	0.061	0.092	0.058	0.037	0.037	0.125	0.048	0.050	0.073	0.094
**CREBBP**	0.489	0.475	0.378	1.000	0.066	0.060	0.098	0.078	0.081	0.093	0.085	0.093	0.072	0.068	0.071	0.073	0.072	0.062	0.118	0.055	0.039	0.026	0.035	0.081	0.044	0.050	0.064	0.065
**DNMT1**	0.097	0.101	0.116	0.066	1.000	0.072	0.165	0.131	0.114	0.156	0.130	0.127	0.126	0.112	0.150	0.109	0.171	0.114	0.064	0.092	0.140	0.047	0.052	0.106	0.068	0.138	0.179	0.145
**EHMT2**	0.070	0.070	0.087	0.060	0.072	1.000	0.092	0.045	0.056	0.094	0.070	0.074	0.072	0.046	0.076	0.071	0.078	0.059	0.047	0.073	0.048	0.072	0.101	0.112	0.081	0.049	0.067	0.106
**EP300**	0.073	0.075	0.098	0.098	0.165	0.092	1.000	0.092	0.089	0.130	0.115	0.111	0.092	0.075	0.122	0.076	0.125	0.070	0.136	0.152	0.099	0.024	0.034	0.097	0.079	0.092	0.114	0.134
**HDAC10**	0.082	0.089	0.098	0.078	0.131	0.045	0.092	1.000	0.591	0.329	0.458	0.444	0.268	0.269	0.342	0.332	0.365	0.294	0.069	0.112	0.097	0.038	0.044	0.072	0.034	0.058	0.095	0.104
**HDAC11**	0.078	0.082	0.085	0.081	0.114	0.056	0.089	0.591	1.000	0.271	0.399	0.348	0.249	0.311	0.291	0.340	0.343	0.305	0.072	0.104	0.084	0.029	0.051	0.061	0.035	0.053	0.094	0.088
**HDAC1**	0.103	0.106	0.146	0.093	0.156	0.094	0.130	0.329	0.271	1.000	0.468	0.442	0.273	0.195	0.496	0.228	0.433	0.246	0.066	0.131	0.129	0.030	0.045	0.104	0.052	0.084	0.139	0.128
**HDAC2**	0.101	0.105	0.130	0.085	0.130	0.070	0.115	0.458	0.399	0.468	1.000	0.562	0.281	0.249	0.363	0.281	0.393	0.280	0.074	0.122	0.125	0.026	0.032	0.089	0.038	0.061	0.126	0.108
**HDAC3**	0.089	0.093	0.124	0.093	0.127	0.074	0.111	0.444	0.348	0.442	0.562	1.000	0.296	0.206	0.420	0.240	0.346	0.258	0.071	0.109	0.105	0.030	0.028	0.090	0.043	0.075	0.120	0.116
**HDAC4**	0.082	0.084	0.107	0.072	0.126	0.072	0.092	0.268	0.249	0.273	0.281	0.296	1.000	0.218	0.318	0.275	0.252	0.273	0.045	0.094	0.089	0.045	0.044	0.076	0.042	0.053	0.067	0.109
**HDAC5**	0.074	0.080	0.085	0.068	0.112	0.046	0.075	0.269	0.311	0.195	0.249	0.206	0.218	1.000	0.194	0.567	0.229	0.488	0.050	0.114	0.099	0.027	0.044	0.061	0.017	0.055	0.100	0.110
**HDAC6**	0.079	0.082	0.118	0.071	0.150	0.076	0.122	0.342	0.291	0.496	0.363	0.420	0.318	0.194	1.000	0.229	0.470	0.258	0.062	0.135	0.124	0.033	0.048	0.082	0.044	0.079	0.117	0.135
**HDAC7**	0.079	0.086	0.093	0.073	0.109	0.071	0.076	0.332	0.340	0.228	0.281	0.240	0.275	0.567	0.229	1.000	0.250	0.483	0.061	0.104	0.082	0.029	0.045	0.046	0.029	0.055	0.084	0.098
**HDAC8**	0.102	0.107	0.132	0.072	0.171	0.078	0.125	0.365	0.343	0.433	0.393	0.346	0.252	0.229	0.470	0.250	1.000	0.278	0.057	0.133	0.114	0.030	0.057	0.101	0.036	0.077	0.112	0.138
**HDAC9**	0.069	0.074	0.090	0.062	0.114	0.059	0.070	0.294	0.305	0.246	0.280	0.258	0.273	0.488	0.258	0.483	0.278	1.000	0.074	0.103	0.098	0.038	0.046	0.064	0.026	0.049	0.102	0.111
**KAT2B**	0.072	0.075	0.061	0.118	0.064	0.047	0.136	0.069	0.072	0.066	0.074	0.071	0.045	0.050	0.062	0.061	0.057	0.074	1.000	0.033	0.071	0.027	0.023	0.040	0.023	0.060	0.082	0.053
**KDM1A**	0.078	0.077	0.092	0.055	0.092	0.073	0.152	0.112	0.104	0.131	0.122	0.109	0.094	0.114	0.135	0.104	0.133	0.103	0.033	1.000	0.113	0.026	0.028	0.074	0.040	0.051	0.095	0.158
**KDM4C**	0.052	0.049	0.058	0.039	0.140	0.048	0.099	0.097	0.084	0.129	0.125	0.105	0.089	0.099	0.124	0.082	0.114	0.098	0.071	0.113	1.000	0.034	0.026	0.058	0.033	0.124	0.129	0.079
**L3MBTL1**	0.036	0.040	0.037	0.026	0.047	0.072	0.024	0.038	0.029	0.030	0.026	0.030	0.045	0.027	0.033	0.029	0.030	0.038	0.027	0.026	0.034	1.000	0.511	0.084	0.023	0.011	0.018	0.024
**L3MBTL3**	0.040	0.040	0.037	0.035	0.052	0.101	0.034	0.044	0.051	0.045	0.032	0.028	0.044	0.044	0.048	0.045	0.057	0.046	0.023	0.028	0.026	0.511	1.000	0.088	0.030	0.014	0.029	0.049
**MAP3K7**	0.106	0.102	0.125	0.081	0.106	0.112	0.097	0.072	0.061	0.104	0.089	0.090	0.076	0.061	0.082	0.046	0.101	0.064	0.040	0.074	0.058	0.084	0.088	1.000	0.060	0.056	0.106	0.078
**MGEA5**	0.041	0.044	0.048	0.044	0.068	0.081	0.079	0.034	0.035	0.052	0.038	0.043	0.042	0.017	0.044	0.029	0.036	0.026	0.023	0.040	0.033	0.023	0.030	0.060	1.000	0.045	0.066	0.074
**NCOA1**	0.026	0.028	0.050	0.050	0.138	0.049	0.092	0.058	0.053	0.084	0.061	0.075	0.053	0.055	0.079	0.055	0.077	0.049	0.060	0.051	0.124	0.011	0.014	0.056	0.045	1.000	0.393	0.063
**NCOA3**	0.051	0.048	0.073	0.064	0.179	0.067	0.114	0.095	0.094	0.139	0.126	0.120	0.067	0.100	0.117	0.084	0.112	0.102	0.082	0.095	0.129	0.018	0.029	0.106	0.066	0.393	1.000	0.083
**PRMT1**	0.088	0.088	0.094	0.065	0.145	0.106	0.134	0.104	0.088	0.128	0.108	0.116	0.109	0.110	0.135	0.098	0.138	0.111	0.053	0.158	0.079	0.024	0.049	0.078	0.074	0.063	0.083	1.000

[a] ECFP4 2048-bit

Table S10. Sequence identity matrix of targets computed from Clustal Omega alignments.

Data set	BRD2	BRD3	BRD4	CREBBP	DNMT1	EHMT2	EP300	HDAC10	HDAC11	HDAC1	HDAC2	HDAC3	HDAC4	HDAC5	HDAC6	HDAC7	HDAC8	HDAC9	KAT2B	KDM1A	KDM4C	L3MBTL1	L3MBTL3	MAP3K7	MGEA5	NCOA1	NCOA3	PRMT1
**BRD2**	1.000	0.653	0.585	0.230	0.134	0.114	0.226	0.081	0.088	0.092	0.075	0.089	0.100	0.121	0.077	0.100	0.087	0.111	0.107	0.093	0.148	0.111	0.097	0.150	0.094	0.131	0.136	0.098
**BRD3**	0.653	1.000	0.602	0.242	0.136	0.098	0.216	0.054	0.110	0.097	0.047	0.089	0.063	0.087	0.082	0.100	0.100	0.075	0.112	0.090	0.140	0.119	0.122	0.180	0.100	0.152	0.116	0.054
**BRD4**	0.585	0.602	1.000	0.238	0.118	0.079	0.217	0.062	0.105	0.098	0.061	0.119	0.077	0.107	0.099	0.101	0.104	0.087	0.095	0.082	0.133	0.115	0.111	0.153	0.093	0.136	0.124	0.077
**CREBBP**	0.230	0.242	0.238	1.000	0.140	0.134	0.637	0.097	0.095	0.078	0.093	0.083	0.147	0.160	0.108	0.120	0.099	0.161	0.125	0.115	0.176	0.143	0.118	0.182	0.102	0.167	0.180	0.078
**DNMT1**	0.134	0.136	0.118	0.140	1.000	0.160	0.133	0.152	0.124	0.118	0.140	0.150	0.146	0.151	0.138	0.138	0.134	0.150	0.153	0.145	0.123	0.158	0.153	0.128	0.141	0.168	0.140	0.197
**EHMT2**	0.114	0.098	0.079	0.134	0.160	1.000	0.135	0.156	0.132	0.125	0.151	0.127	0.192	0.192	0.169	0.175	0.132	0.187	0.151	0.180	0.122	0.180	0.144	0.077	0.198	0.138	0.141	0.107
**EP300**	0.226	0.216	0.217	0.637	0.133	0.135	1.000	0.104	0.108	0.078	0.089	0.076	0.155	0.167	0.117	0.131	0.090	0.161	0.102	0.128	0.188	0.139	0.119	0.176	0.120	0.164	0.148	0.079
**HDAC10**	0.081	0.054	0.062	0.097	0.152	0.156	0.104	1.000	0.202	0.247	0.851	0.591	0.240	0.235	0.231	0.223	0.405	0.249	0.106	0.156	0.099	0.113	0.116	0.067	0.175	0.130	0.124	0.140
**HDAC11**	0.088	0.110	0.105	0.095	0.124	0.132	0.108	0.202	1.000	0.240	0.187	0.179	0.376	0.353	0.416	0.375	0.198	0.393	0.116	0.132	0.106	0.141	0.110	0.135	0.137	0.174	0.124	0.115
**HDAC1**	0.092	0.097	0.098	0.078	0.118	0.125	0.078	0.247	0.240	1.000	0.246	0.228	0.230	0.224	0.262	0.224	0.231	0.227	0.123	0.133	0.097	0.114	0.119	0.105	0.126	0.124	0.109	0.112
**HDAC2**	0.075	0.047	0.061	0.093	0.140	0.151	0.089	0.851	0.187	0.246	1.000	0.591	0.225	0.236	0.237	0.208	0.407	0.252	0.117	0.156	0.102	0.112	0.120	0.067	0.150	0.126	0.123	0.127
**HDAC3**	0.089	0.089	0.119	0.083	0.150	0.127	0.076	0.591	0.179	0.228	0.591	1.000	0.229	0.239	0.214	0.208	0.410	0.234	0.134	0.181	0.081	0.136	0.114	0.062	0.133	0.123	0.112	0.179
**HDAC4**	0.100	0.063	0.077	0.147	0.146	0.192	0.155	0.240	0.376	0.230	0.225	0.229	1.000	0.616	0.353	0.534	0.224	0.597	0.146	0.210	0.136	0.152	0.096	0.112	0.153	0.156	0.108	0.103
**HDAC5**	0.121	0.087	0.107	0.160	0.151	0.192	0.167	0.235	0.353	0.224	0.236	0.239	0.616	1.000	0.342	0.483	0.218	0.611	0.121	0.198	0.136	0.138	0.113	0.103	0.148	0.137	0.109	0.108
**HDAC6**	0.077	0.082	0.099	0.108	0.138	0.169	0.117	0.231	0.416	0.262	0.237	0.214	0.353	0.342	1.000	0.335	0.220	0.352	0.130	0.171	0.130	0.140	0.103	0.121	0.138	0.163	0.141	0.109
**HDAC7**	0.100	0.100	0.101	0.120	0.138	0.175	0.131	0.223	0.375	0.224	0.208	0.208	0.534	0.483	0.335	1.000	0.212	0.482	0.145	0.186	0.141	0.132	0.112	0.129	0.160	0.149	0.104	0.115
**HDAC8**	0.087	0.100	0.104	0.099	0.134	0.132	0.090	0.405	0.198	0.231	0.407	0.410	0.224	0.218	0.220	0.212	1.000	0.229	0.114	0.171	0.096	0.142	0.137	0.113	0.145	0.131	0.115	0.108
**HDAC9**	0.111	0.075	0.087	0.161	0.150	0.187	0.161	0.249	0.393	0.227	0.252	0.234	0.597	0.611	0.352	0.482	0.229	1.000	0.146	0.197	0.134	0.145	0.102	0.120	0.149	0.148	0.117	0.137
**KAT2B**	0.107	0.112	0.095	0.125	0.153	0.151	0.102	0.106	0.116	0.123	0.117	0.134	0.146	0.121	0.130	0.145	0.114	0.146	1.000	0.145	0.084	0.133	0.156	0.146	0.134	0.164	0.154	0.135
**KDM1A**	0.093	0.090	0.082	0.115	0.145	0.180	0.128	0.156	0.132	0.133	0.156	0.181	0.210	0.198	0.171	0.186	0.171	0.197	0.145	1.000	0.083	0.118	0.117	0.111	0.114	0.138	0.126	0.116
**KDM4C**	0.148	0.140	0.133	0.176	0.123	0.122	0.188	0.099	0.106	0.097	0.102	0.081	0.136	0.136	0.130	0.141	0.096	0.134	0.084	0.083	1.000	0.132	0.133	0.154	0.112	0.134	0.122	0.084
**L3MBTL1**	0.111	0.119	0.115	0.143	0.158	0.180	0.139	0.113	0.141	0.114	0.112	0.136	0.152	0.138	0.140	0.132	0.142	0.145	0.133	0.118	0.132	1.000	0.442	0.135	0.120	0.141	0.133	0.106
**L3MBTL3**	0.097	0.122	0.111	0.118	0.153	0.144	0.119	0.116	0.110	0.119	0.120	0.114	0.096	0.113	0.103	0.112	0.137	0.102	0.156	0.117	0.133	0.442	1.000	0.144	0.125	0.179	0.145	0.093
**MAP3K7**	0.150	0.180	0.153	0.182	0.128	0.077	0.176	0.067	0.135	0.105	0.067	0.062	0.112	0.103	0.121	0.129	0.113	0.120	0.146	0.111	0.154	0.135	0.144	1.000	0.072	0.133	0.119	0.068
**MGEA5**	0.094	0.100	0.093	0.102	0.141	0.198	0.120	0.175	0.137	0.126	0.150	0.133	0.153	0.148	0.138	0.160	0.145	0.149	0.134	0.114	0.112	0.120	0.125	0.072	1.000	0.148	0.140	0.063
**NCOA1**	0.131	0.152	0.136	0.167	0.168	0.138	0.164	0.130	0.174	0.124	0.126	0.123	0.156	0.137	0.163	0.149	0.131	0.148	0.164	0.138	0.134	0.141	0.179	0.133	0.148	1.000	0.391	0.141
**NCOA3**	0.136	0.116	0.124	0.180	0.140	0.141	0.148	0.124	0.124	0.109	0.123	0.112	0.108	0.109	0.141	0.104	0.115	0.117	0.154	0.126	0.122	0.133	0.145	0.119	0.140	0.391	1.000	0.110
**PRMT1**	0.098	0.054	0.077	0.078	0.197	0.107	0.079	0.140	0.115	0.112	0.127	0.179	0.103	0.108	0.109	0.115	0.108	0.137	0.135	0.116	0.084	0.106	0.093	0.068	0.063	0.141	0.110	1.000
